# Bone metastasis risk and prognosis assessment models for kidney cancer based on machine learning

**DOI:** 10.3389/fpubh.2022.1015952

**Published:** 2022-11-17

**Authors:** Lichen Ji, Wei Zhang, Jiaqing Huang, Jinlong Tian, Xugang Zhong, Junchao Luo, Senbo Zhu, Zeju He, Yu Tong, Xiang Meng, Yao Kang, Qing Bi

**Affiliations:** ^1^Cancer Center, Department of Orthopedics, Zhejiang Provincial People's Hospital, Affiliated People's Hospital, Hangzhou Medical College, Hangzhou, Zhejiang, China; ^2^Department of Laboratory Medicine, Zhejiang Provincial People's Hospital, Affiliated People's Hospital, Hangzhou Medical College, Hangzhou, Zhejiang, China; ^3^Center for Rehabilitation Medicine, Osteoporosis Center, Zhejiang Provincial People's Hospital (Affiliated People's Hospital, Hangzhou Medical College), Hangzhou, Zhejiang, China; ^4^Department of Orthopedics, The Second Affiliated Hospital and Yuying Children's Hospital of Wenzhou Medical University, Wenzhou, China; ^5^Department of Orthopedics, Zhejiang Provincial People's Hospital, Qingdao University, Qingdao, China; ^6^The Second Clinic Medical College, Zhejiang Chinese Medicine University, Hangzhou, China; ^7^The First Affiliated Hospital of Bengbu Medical University, Bengbu, Anhui, China

**Keywords:** kidney cancer, bone metastasis, diagnosis, prognosis, machine learning, predicting model

## Abstract

**Background:**

Bone metastasis is a common adverse event in kidney cancer, often resulting in poor survival. However, tools for predicting KCBM and assessing survival after KCBM have not performed well.

**Methods:**

The study uses machine learning to build models for assessing kidney cancer bone metastasis risk, prognosis, and performance evaluation. We selected 71,414 kidney cancer patients from SEER database between 2010 and 2016. Additionally, 963 patients with kidney cancer from an independent medical center were chosen to validate the performance. In the next step, eight different machine learning methods were applied to develop KCBM diagnosis and prognosis models while the risk factors were identified from univariate and multivariate logistic regression and the prognosis factors were analyzed through Kaplan-Meier survival curve and Cox proportional hazards regression. The performance of the models was compared with current models, including the logistic regression model and the AJCC TNM staging model, applying receiver operating characteristics, decision curve analysis, and the calculation of accuracy and sensitivity in both internal and independent external cohorts.

**Results:**

Our prognosis model achieved an AUC of 0.8269 (95%CI: 0.8083–0.8425) in the internal validation cohort and 0.9123 (95%CI: 0.8979–0.9261) in the external validation cohort. In addition, we tested the performance of the extreme gradient boosting model through decision curve analysis curve, Precision-Recall curve, and Brier score and two models exhibited excellent performance.

**Conclusion:**

Our developed models can accurately predict the risk and prognosis of KCBM and contribute to helping improve decision-making.

## Introduction

It is estimated that kidney cancer (KC) accounts for the 16th most common malignant tumor in the world (1). Based on the NCCN Clinical Practice Guidelines in Oncology, 76,080 Americans were diagnosed with KC, and 13,780 died in 2021 because of these diseases (2). KC incidence displayed a continuous upward trend before the 1990s but has remained stable or declined in many countries (3, 4). Clear cell renal cell carcinoma (ccRCC), papillary, and chromophobe are the most common subtypes, accounting for 85–90% of all primary KC (5, 6). A study found that 75% of KC patients survived more than 5 years after diagnosis. However, once metastases were found, the 5-year survival rate of the patient dropped to 12% (2).

As a result, cancer metastasis to vital distant organs is considered the final phase of cancer progression, which involves a series of stochastic events known as the metastatic cascade (7, 8). A study by M.Bianchi found the proportions of the most common metastasis site in KC are 45.2% in lung, 29.5% in bone, 21.8% in lymph node, 20.3% in liver, 8.9% in adrenal, and 8.1% in brain (9). Instead of primary tumors, we focus on bone metastases (BM) because they are responsible for 90% of all cancer death (10). BM also causes complications, including pain, hypercalcemia, anemia, inflammation, skeletal fractures, spinal cord compression, instability, and decreased mobility (11–13).

Each of these complications compromises the quality of life and the functional status. The prognosis for kidney cancer with bone metastases (KCBM) remains uncertain, despite advances in surgery, radiation therapy, and targeted medical therapy developed to treat BM (14). In KCBM, most therapies are used to improve skeletal adverse events, not to lower BM rates (15).

There is a dire need to find a standard treatment guideline to reduce the occurrence of KCBM and improve the survival rate among KCBM patients. Furthermore, we need reliable predicting tools to assess the probability of events. Numerous works of literature have developed several nomograms to predict prognosis or evaluate the diagnosis risk of KCBM (16, 17). Nevertheless, we believe the two risk estimation models can improve performance in new ways. Recently, some scholars have used new algorithms to establish bone metastasis diagnosis models of kidney cancer. Due to the huge data in the SEER database and the scientific nature of the new algorithm, the model performance has been improved. However, the rationality and completeness of the included variables still need to be improved, and the performance evaluation also needs to be supplemented (18).

A growing number of these methods named, artificial intelligence, or machine learning, have now been put in use for biomedical research (19). The application of machine learning can promote the overall quality of prediction in a wide range of practical applications through high-throughput training and taking an ensemble learning approach. Several models can be used to predict the risk of KCBM, including logistic regression (LR), naive Bayes BS classifier (NBC), decision tree (DT), extreme gradient boosting (XGBoost), multilayer perceptron (MLP), random forest (RF), support vector machine (SVM), and k-nearest neighbor (KNN) (20–24). Our study aims to develop several models and compare their functions using different methods of operation. Then we apply the models with the highest performance to clinical valuation and they should provide a more accurate diagnosis and prognosis of KCBM and can help develop treatment guidelines and standard treatment for KCBM.

## Materials and methods

### Study design and participants

We extracted patients with KC diagnosed from 2010 to 2016 in the SEER database, considered the most common and authoritative cancer database in the USA. The inclusion criteria included: (1) kidney cancer patients with complete survival data; (2) the ensured effectiveness of follow-up; (3) the source of the case should exclude all cases obtained through necropsy and maintain those determined on the death report; (4) KC diagnosed by pathology, alone with BM observed by imaging or pathology.

**Figure 1:**
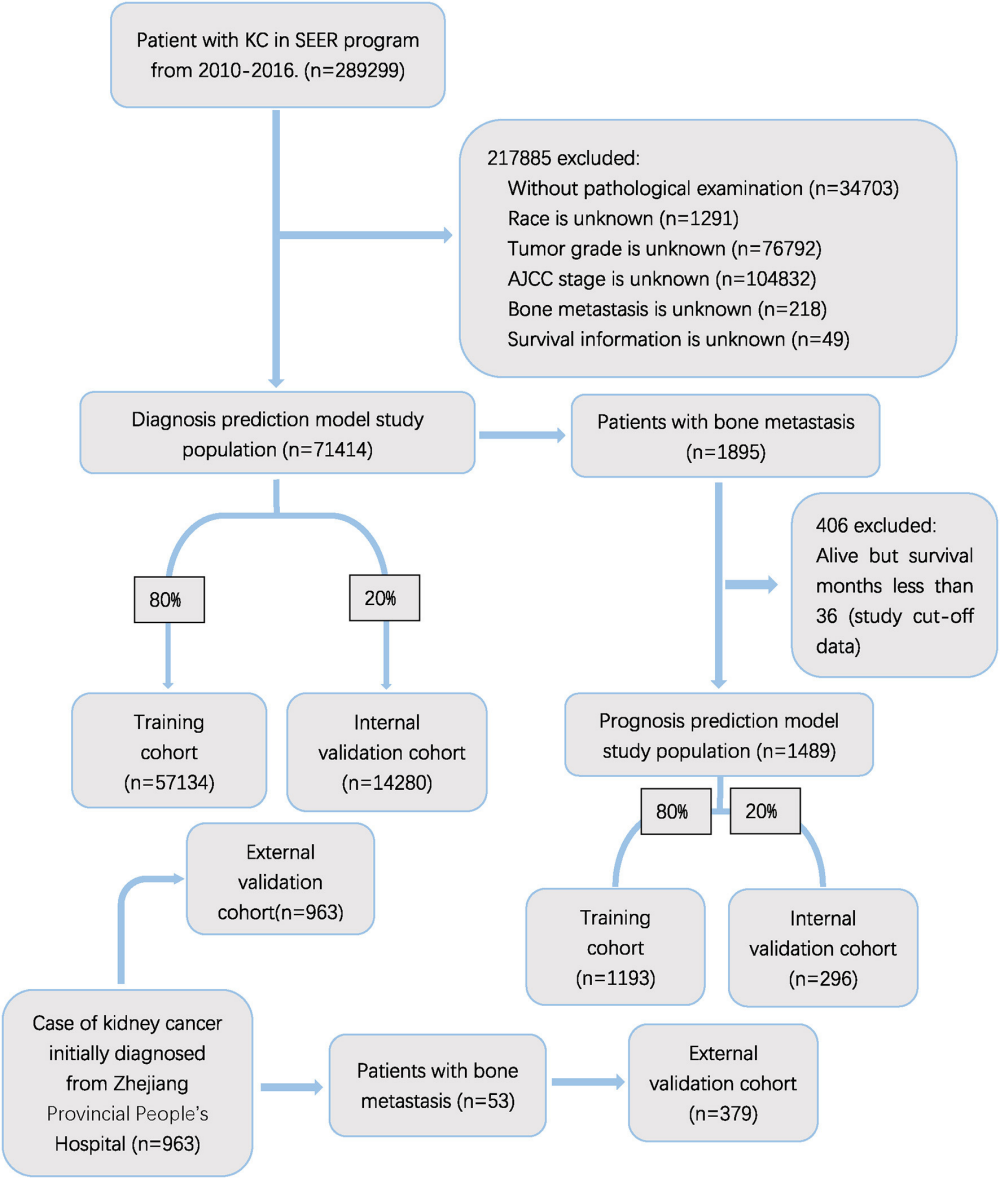
Flow diagram of the study population selected from SEER database and the Zhejiang Provincial People's Hospital. According to the inclusion and exclusion criteria, a total of 71,414 patient were included in this study,and they were randomly cut into the training and internal test sets in a 8:2 ratio. Data from the Zhejiang provincial People's Hospital as an external test set (965 patients).

The exclusion criteria involve: (1) unavailable clinical or relevant examination information, (2) unknown survival information. The flow chart of the study is shown in Figure 1.

### Data collection

The selected baseline characteristics were age at diagnosis, race, gender, primary site, grade, histology subtype, marital status, insurance recode, stage, TNM stage, surgery, lymph node surgery, radiation recode, chemotherapy, brain metastasis, liver metastasis, lung metastasis, laterality, and tumor size. Furthermore, histological type code were divided into seven group based on the International Classification of Disease for Oncology (ICD-O): transitional cell carcinoma (8120), papillary transitional cell carcinoma (8130), papillary adenocarcinoma (8260), clear-cell adenocarcinoma (8310), renal cell carcinoma (8312), renal cell carcinoma chromophobe type (8317), other types (8000, 8005, 8010, 8012, 8013, 8020, 8022, 8031–8033, 8035, 8041, 8045, 8046, 8050, 8052, 8070–8072, 8074, 8082, 8083, 8121, 8122, 8130, 8131, 8140, 8210, 8211, 8240, 8246, 8249, 8255, 8250, 8263, 8270, 8280, 8290, 8310, 8312, 8313, 8315–8319, 9320, 8323, 8330, 8342, 8480, 8481, 8490, 8510, 8522, 8560, 8574, and 8980). Oncology staging was determined on the basis of the 7th TNM classification of the AJCC. Regarding the survival model of KCBM, overall survival (OS) was seen as the primary endpoint event.

### Statistical analysis

In this study, R (Version 4.1.3), IBM SPSS Statistics (Version 22), and Python (Version 3.9.7) were utilized to complete all statistical analyses. We used the following package: “foreign,” ”survival,” “caret,” ”rms,” “survminer,”” sklearn.linear_model,”“ sklearn.ensemble” “ sklearn.tree” “ sklearn.svm” “ sklearn.neural_network ” “ sklearn.tree.” The specific code has been uploaded to guthub (https://github.com/JiLichen/Kidney-diagnosis-and-prognosis). All KC patients were divided into a training and a validation cohort at random according to the proportion of 8:2. Continuous data was compared by independent sample *t*-tests or Mann-Whitney U tests and categorical data was compared by chi-square tests or Fisher exact test. All variables we included in the analysis were analyzed by univariate logistic regression. Values of *p* < 0.05 were regarded statistically significant. Multivariate logistic regression was utilized to test whether or not these significant factors were associated with BM in patients with KC. To determine independent prognostic factors of KCBM, the cohort with BM were grouped by an 8:2 ratio as training and validation cohorts. Then, Kaplan-Meier analysis and Cox proportional hazard regression analysis were conducted on the factors above and *p* < 0.05 were regarded statistically significant. Aiming to develop a model to predict the risk and overall survival of KCBM accurately, we used LR, NBC, DT, XGBoost, MLP, RF, SVM, and KNN algorithms based on the risk factors to establish diagnosis models. Additionally, we applied the above algorithms to independent prognostic factors to build surviving models at the 3-year observation point. After testing the various performances of the above two types of models, we selected the most representative models as clinical recommendations (25, 26).

We use the following formula to calculate the performance of the model:


(1) $$\begin{array}{l} Accuracy=\frac{TP+TN}{TP+FN+TN+FN} \end{array}$$

(2) $$\begin{array}{l} Precision=\frac{TP}{TP+FP} \end{array}$$

(3) $$\begin{array}{l} Sensitivity=\frac{TP}{TP+FN}\;=recall \end{array}$$

(4) $$\begin{array}{l} F1=\frac{2^{*}P^{*}R}{P+R} \end{array}$$

(5) $$\begin{array}{l} Brierscore=\frac{1}{N}\overset{N}{\underset{T=1}{\sum }}\left(f_{t}-o_{t}\right) \end{array}$$

### Model visualization

We used web pages to establish risk assessment tools for diagnosis and prognosis in kidney cancer patients. Clinicians can log into the website to utilize the risk assessment tools.

## Results

### Cohort description

Our study included 71,414 patients from SEER database after above screening. As a result, 1,895 (2.6%) patients were observed to have KCBM. In the cohort of patients with BM, 406 patients who was alive but follow-up time <36 were excluded. 1,385 (93.0%) of the remaining 1,489 patients died during an average of 35.74 months (Std = 22.33) of follow-up. The training cohort comprised 1,193 and remaining 296 patients formed an internal validation cohort according to the grouping ratio of 8:2, respectively. 71,414 patients of diagnose cohort was grouped in the same way. The randomness of the grouping was verified by the chi-square test and *t*-test (Supplementary Tables 1, 4). The 963 patients from Zhejiang Provincial People's Hospital were selected as an external validation cohort, with 53 patients have KCBM. Details of variables including sociodemographic characteristics, clinical features and treatment regimens of kidney cancer patients are demonstrated in Supplementary Table 2.

The distribution and characteristics of KC group and KCBM group are shown in Supplementary Tables 2, 4. Elderly people aged 60–69 are the most common in training cohort (31.1%). Males with KC nearly twice as many as women (63.7%). Clear-cell adenocarcinoma accounts for the largest proportion among all histology types (59.7%). The commonest grade, T and *n* stage are grade II (47.4%), T1 (64.2%) and N0 (93.8%). The primary lesions are basically equal to the left and right in laterality (49.4 in left and 50.4% in right). In terms of treatment, the vast majority of patients have undergone different types of surgery. Among them, radical nephrectomy account for about half, reaching 44.5%. A small number of patients of patients underwent lymph node removed surgery (13.3%). Additionally, 1,476 (2.5%) patients were treated with radiation therapy and 4,299 (7.5%) patients received chemotherapy. Regarding the distance metastasis of KC, 2,607 (4.5%) patients were detected to have lung metastasis, 786 (1.3%) patients had liver metastasis and 392 (0.6%) had brain metastasis.

In external validation cohort, due to geographical restriction, the included cases are all Asian. Elder people age 50–69 accounts for 51.4% of all age groups. There are also about twice as many male patients as female patients. Clear-cell adenocarcinoma is the most common histology type (85.7%). Radical nephrectomy (30.7%) and partial/ subtotal nephrectomy/ partial ureterectomy (45.6%) have the largest proportion and 56 patients (5.8%) accept lymph nodes removed surgical treatment. Regarding other treatments, 15 patients (1.5%) received radiation therapy, 64 patients (6.6%) were treated with chemotherapy. The metastatic status of distant organs is shown as follows: lung metastasis (3.2%), liver metastasis (1.4%), brain metastasis (0.5%).

### Independent risk factors for KCBM

According to Supplementary Table 3, we selected variables about sociodemographic characteristics, clinical features, treatment regimens and performed a univariate logistic regression analysis on them. The result demonstrated that nineteen variables met the requirement of *p* < 0.05. Furthermore, the variables mentioned above were selected through multivariable logistic regression. Independent predictors of KCBM contained age, primary site, grade, histology, *n* stage, surgery, radiation therapy, chemotherapy, brain metastasis, liver metastasis, lung metastasis and laterality. The correlation analysis was performed with Spearman correlation coefficient test and revealed no significant correlation between variables we included in diagnosis models (Supplementary Figure 1).

### Diagnosis machine learning model construction

Based on the independent predictors screened through logistic regression, we developed eight models using machine learning methods in the training cohort, such as decision tree (DT), random forest (RF), multilayer perceptron (MLP), logistic regression (LR), naive Bayes BS classifier (NBC), extreme gradient boosting (XGB), support vector machine (SVM) and k-nearest neighbor (KMN).

### Diagnostic machine learning model validation

**Figure 2:**
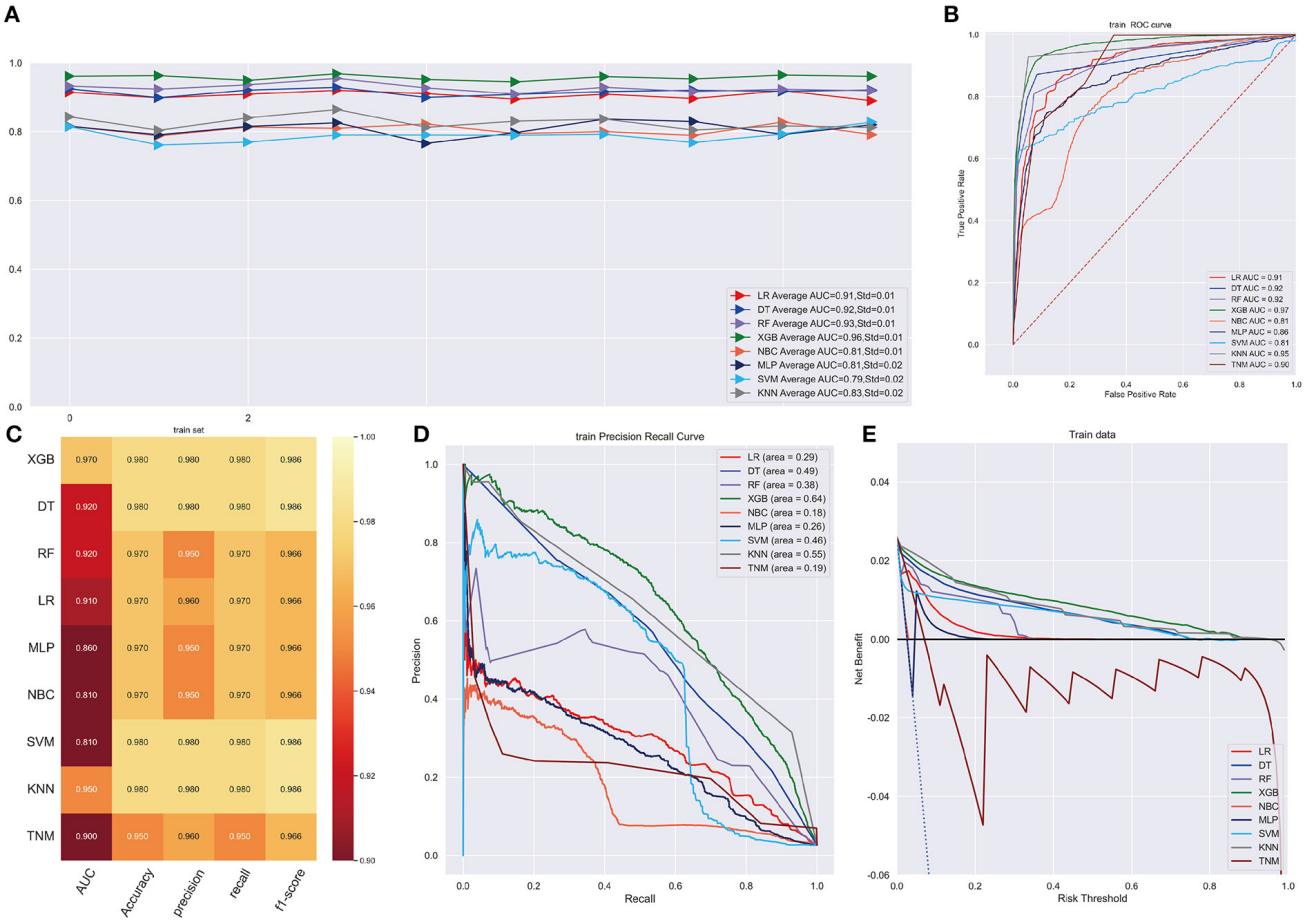
**(A)** Ten-fold cross-validation results of different machine models in training set. LR, Logistic regression; DT, Decision tree; RF, Random Forest; XGB, eXtreme gradient boosting; NBC, Naive Bayes classification; MLP, Multilayer Pecepreon; SVM, support vector machine; KMN, k-nearest neighbor. **(B)** The ROC curve of different machine learning models in training test set. **(C)** Prediction performance of different models in training set. **(D)** The PR curve of different machine learning models in training test set. **(E)** The DCA curve of different machine learning models in training test set.

As illustrated in Figure 2A, the XGB model performed well in ten-cross validations with an average AUC of 0.96 (Std = 0.01) while LR model, DT model, RF model, NBC model, MLP model, SVM model, KMN model indicated average AUC of 0.91 (Std = 0.01), 0.92 (Std = 0.01), 0.93 (Std = 0.01), 0.81 (Std = 0.01), 0.81 (Std = 0.02), 0.79 (Std = 0.02), 0.83 (Std = 0.02). The discrimination performance of different machine learning models was evaluated by receiver operating characteristic (ROC) curve analysis and XGB model had a highest AUC of 0.97 (95%CI: 0.9469–0.9817) (Figure 2B). Additionally, XGB model achieve a Brier score of 0.014, which was lower than that Brier scores of LR model (0.022), DT model (0.016), RF model (0.019), NBC model (0.025), MLP model (0.025), SVM model (0.018), KMN model (0.015). Other performances were shown in Figure 2C. PR curve and DCA curve were performed to test the models in training cohort and XGB model was proved to be highly reliable (Figures 2D,E).

**Figure 3:**
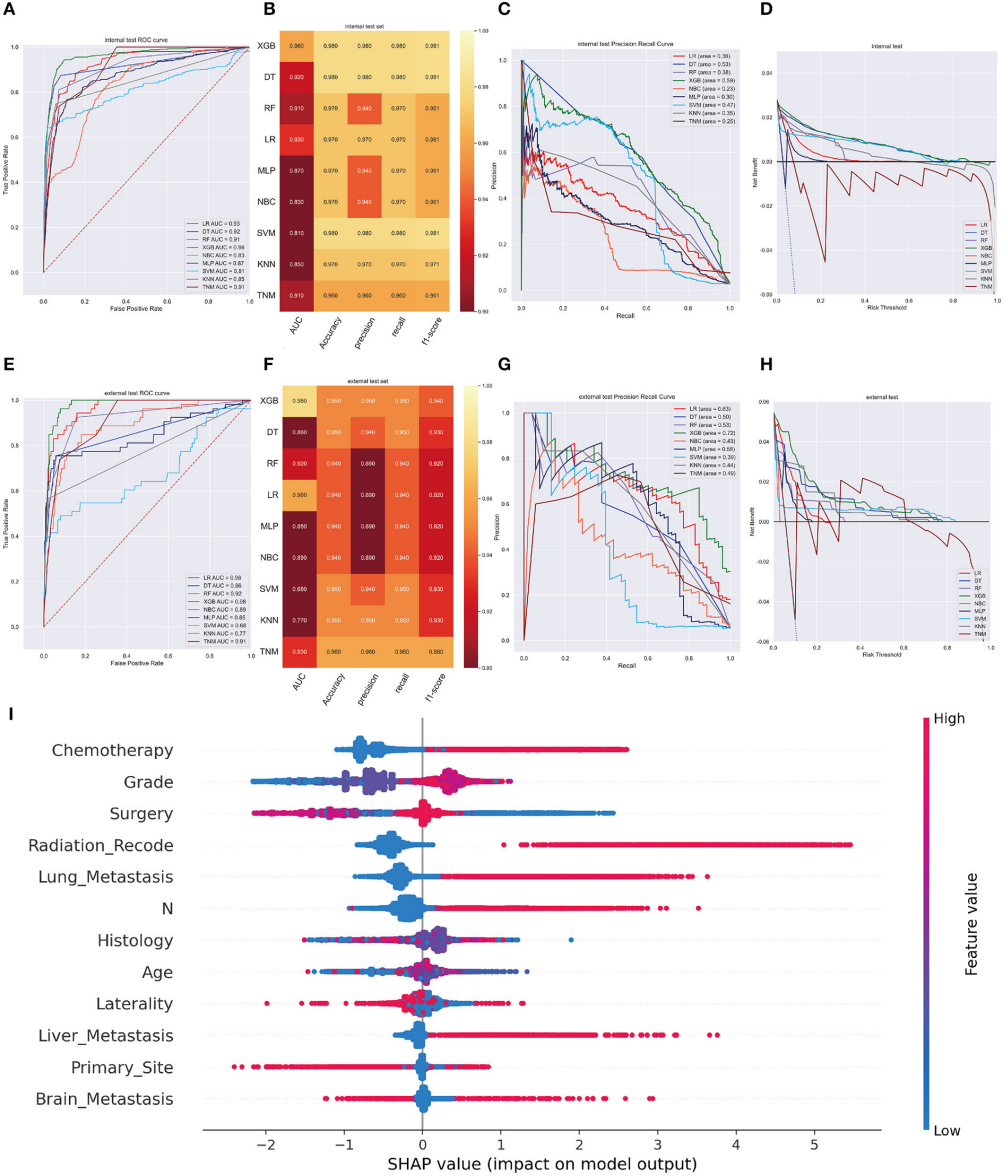
**(A)** The ROC curve of different machine learning models in internal test set. **(B)** Prediction performance of different models in internal test set. **(C)** The PR curve of different machine learning models in internal test set. **(D)** The DCA curve of different machine learning models in internal test set. **(E)** The ROC curve of different machine learning models in external test set. **(F)**. Prediction performance of different models in external test set. **(G)** The PR curve of different machine learning models in external test set. **(H)** The DCA curve of different machine learning models in external test set. **(I)** Summary plots for SHAP values. For each feature, one point corresponds to a single patient. A point's position along the x axis (i.e., the actual SHAP value) represents the impact that feature had on the model's output for that specific patient. (diagnosis model).

XGB model achieved the best AUC of 0.960 (accuracy of 0.980, precision of 0.980, sensitivity of 0.980, f1-score of 0.981) in internal validation cohort and got an AUC of 0.980 (accuracy of 0.950, precision of 0.950, sensitivity of 0.950, f1-score of 0.940) in external validation cohort (Figures 3A,B,E,F). Moreover, Figures 3C,G shows that the area under the PR curve for the XGB is larger than any other model, including the TNM staging model. To further validate the potency of the model in clinical practice, the DCA curve depicted that the XGB model still performed well (Figures 3D,H).

In the SHAP graph, each point represents a single patient for each feature in the XGB model. The location of a point along the actual SHAP value corresponds the effect a variable had on output of the model for that case (Figure 3I). Moreover, Supplementary Figure 2 showed the feature importance in each algorithm and Supplementary Figure 3 illustrated the prediction results of the models as a heatmap.

### Characteristics and survival analyses of KCBM

Supplementary Figure 4A displays that the overall survival curve for 1489 KCBM patients we selected from the SEER database declined rapidly before the 3-year cut-off while the curve declined slowly after the 3-year time point. As a result, selecting 3 years as the predicting node has a high clinical value for treatment planning. Clinical features and treatment regimens information of KC patients with BM are displayed in Supplementary Table 5. Using a ratio of 8:2, the study population was randomly split into a training set and a validation set. The Chi-square test and Fisher's exact test results showed that there were no significant differences in the characteristics between the training cohort and validation cohort (Supplementary Table 4). When the Kaplan-Meier survival curves and log-rank tests were used on categorical variables, it was discovered that characteristics including race (p = 0.47), insurance (p = 0.980), and lymph node surgery (p = 0.44) were not thought to have a sufficient influence on survival. Age (p < 0.001), sex (p = 0.037), primary site (p < 0.001), grade (p < 0.001), histology (p < 0.001), marital status (p < 0.001), T stage (p < 0.001), *n* stage (p < 0.001), surgery (p < 0.001), radiation therapy (p = 0.001), chemotherapy (p = 0.002), brain metastasis (p < 0.001), liver metastasis (p < 0.001), lung metastasis (p < 0.001), and laterality (p=0.002) affected survival significantly (Supplementary Figures 4B–P). The continuous variable such as tumor size was evaluated using Cox proportional hazard regression analysis and it was closely related to patient survival (HR 1.001, 95%CI 1.000–1.001, *p* < 0.001). Lastly, it was found that age, sex, primary site, grade, histology subtype, marital status, T stage, *n* stage, surgery, chemotherapy, radiation therapy, brain metastasis, liver metastasis, lung metastasis, laterality and tumor size were tested as independent prognostic factors for OS and these variables were selected into model construction (Supplementary Table 6). A correlation analysis was used to interpret the independence of each selected variable (Supplementary Figure 5).

### Prognostic machine learning model development and validation

**Figure 4:**
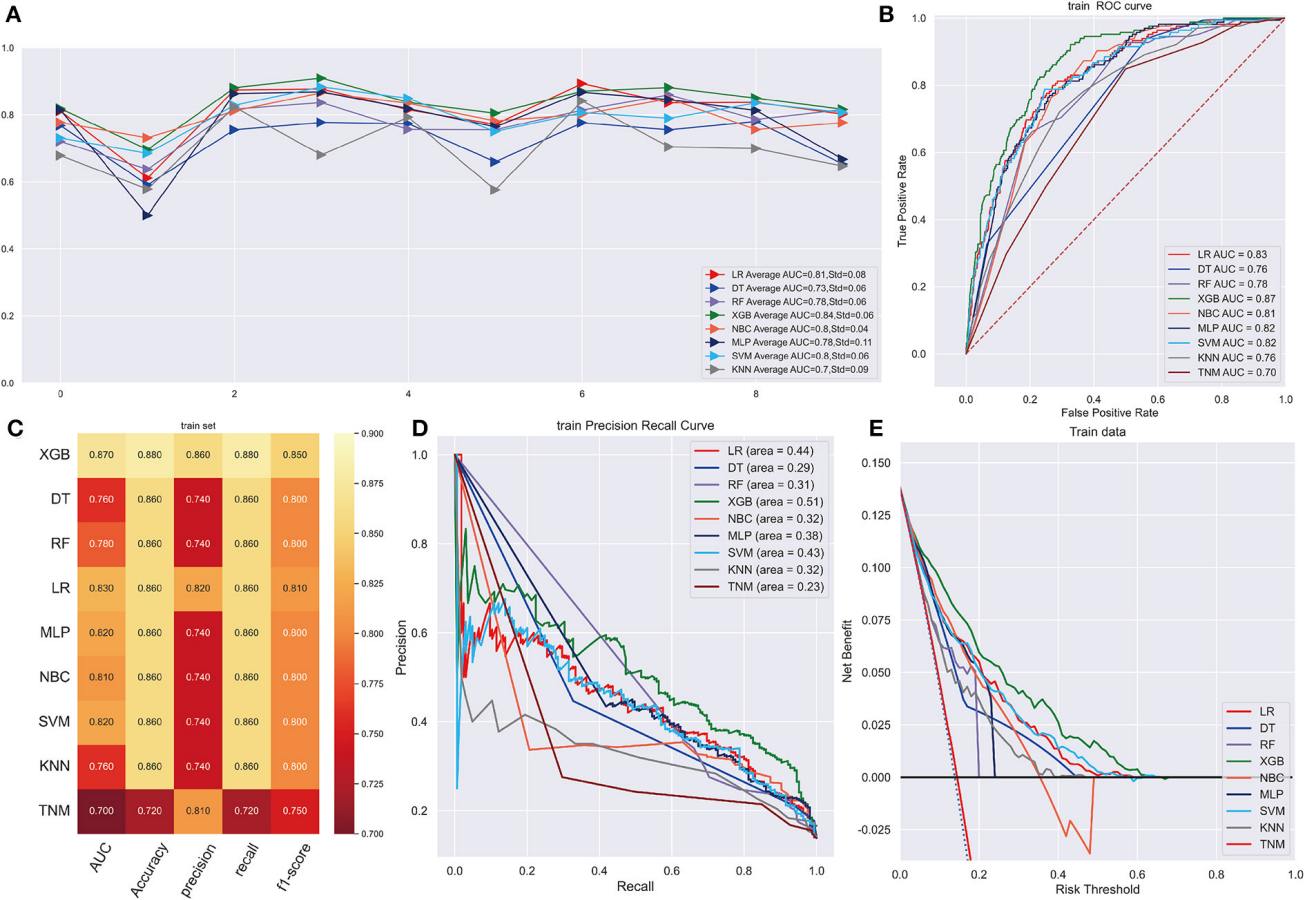
**(A)** Ten-fold cross-validation results of different machine models in training set. LR, Logistic regression; DT, Decision tree; RF, Random Forest; XGB, eXtreme gradient boosting; NBC, Naive Bayes classification; MLP, Multilayer Pecepreon; SVM, support vector machine; KMN, k-nearest neighbor. **(B)** The ROC curve of different machine learning models in training test set. **(C)** Prediction performance of different models in training set. **(D)** The PR curve of different machine learning models in training test set. **(E)** The DCA curve of different machine learning models in training test set.

**Figure 5:**
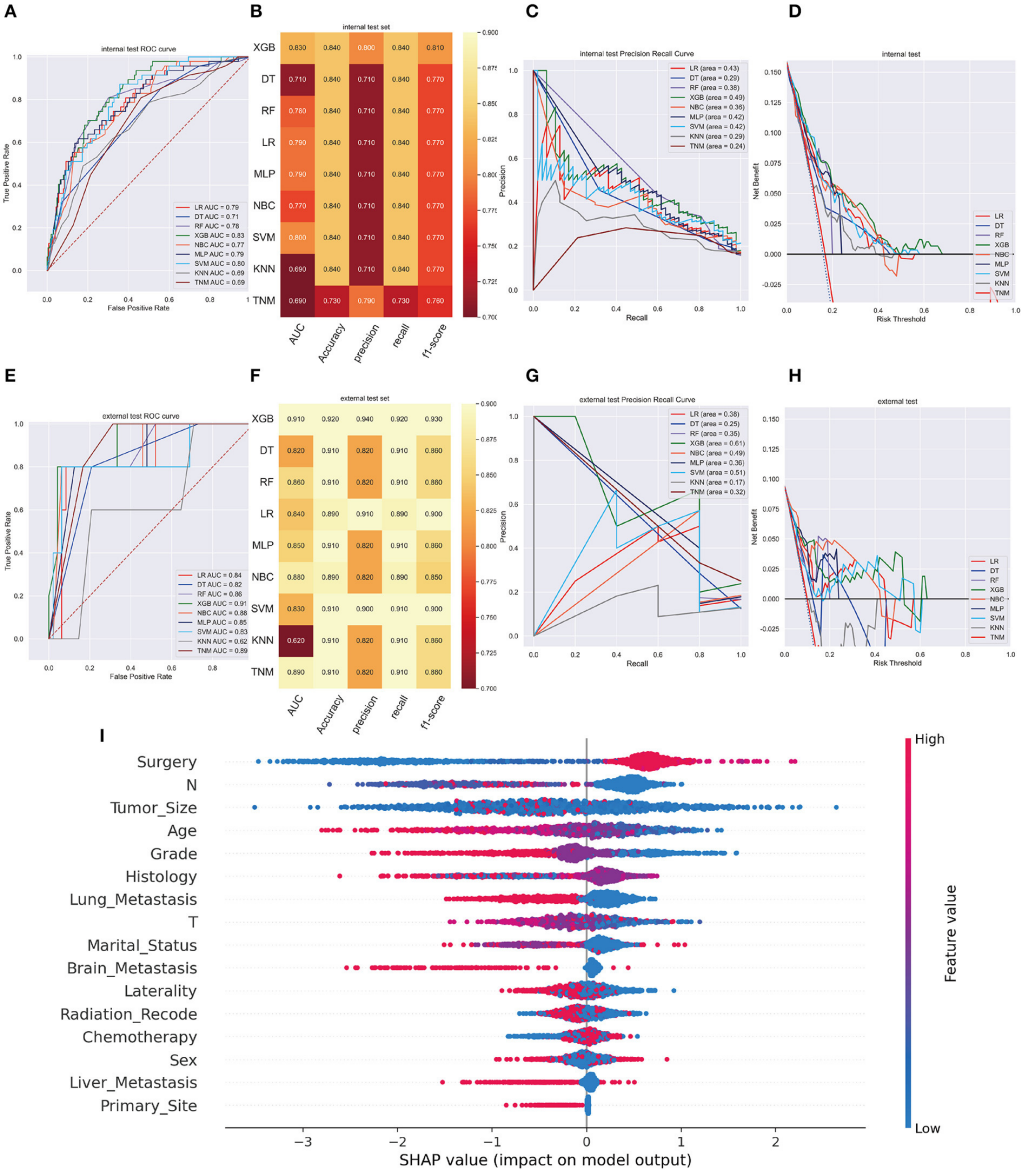
**(A)** The ROC curve of different machine learning models in internal test set. **(B)** Prediction performance of different models in internal test set. **(C)** The PR curve of different machine learning models in internal test set. **(D)** The DCA curve of different machine learning models in internal test set. **(E)** The ROC curve of different machine learning models in external test set. **(F)** Prediction performance of different models in external test set. **(G)** The PR curve of different machine learning models in external test set. **(H)** The DCA curve of different machine learning models in external test set. **(I)** Feature importance plot for the XGB prognosis prediction model. All the features are shown in this figure. The blue and red points in each row represent nodules having low to high values of the specific feature, while the *x*-axis shows the SHAP value, indicating the impact on the model. (prognosis model).

Following data splitting, patients were used for training and ten-fold cross-validations of the algorithms while validation was performed using the remaining 296 patients. Figure 4A shows that XGB got a highest average AUC of 0.84 (Std = 0.06), predicting 3-year OS in the training cohort. The AUC values were 0.87 (95%CI: 0.8499–0.8824) for the prediction of 3-year OS indicating the model had a superior predictive ability. AUC of other machine learning models and TNM staging is listed in Figure 4B. In internal and external validation cohort, our prognosis model got AUCs of 0.83 (95%CI: 0.8083–0.8425) and 0.91 (95%CI: 0.8979–0.9261), respectively (Figures 5A,E). We calculated the integrated Brier score to assess the accuracy of the established models and the results were as follows, XGB: 0.088; DT: 0.103; RF: 0.110; LR: 0.097; MLP: 0.104; NBC: 0.104; SVM: 0.097; KMN: 0.107. The metrics of each machine learning algorithm on these datasets are demonstrated in Figures 4C, 5B,F. Furthermore, the area under PR curve reached 0.51 in training cohort, 0.49 in the internal validation cohort, and 0.61 in the external validation cohort (Figures 4D, 5C,G). DCA suggests net benefits of the eight different models and TNM staging as displayed in Figures 4E, 5D,H. We estimated each feature's impact on predicting prognosis in the XGB model by observing the SHAP values (Figure 5I). The feature importance of each model was shown in Supplementary Figure 6. The predicted results of the XGB model and the TNM staging in training and validation cohort are listed on the heatmap (Supplementary Figure 7).

### Web predictor

To contribute to clinical decision-making, we developed two web-based calculators based on XGB machine learning algorithm for KCBM diagnosis and prognosis prediction. The website addresses were as follows:

https://share.streamlit.io/lry4000/sa/main;

https://share.streamlit.io/lryoxidkghwqls/survival_three_years/main.

**Figure 6:**
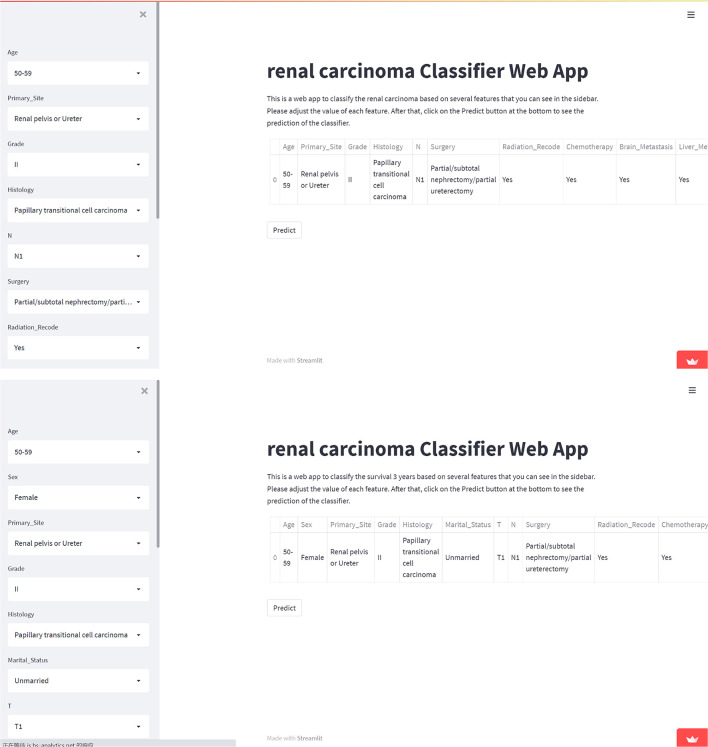
Screenshot of the web-based model. Screenshot of the XGB KCBM diagnosis and prognosis predicting model, which is available at https://share.streamlit.io/lry4000/sa/main; https://share.streamlit.io/lryoxidkghwqls/survival_three_years/main.

Users can directly enter variable values and estimate the probability of occurrence and survival of KCBM. A snapshot of the online calculator is demonstrated in Figure 6.

## Discussion

One of the most frequent locations for the spread of hematogenous tumor cells in KC is the bone. At advanced illness stages, patients with KC are more prone to develop painful and functionally incapacitating bone metastases (27). Furthermore, approximately one-third of patients with metastasis KC have already been diagnosed with bone metastasis, and another third of patients will develop them during their course of the disease (28). In our study, we found that the survival rate of patients with KCBM decreased sharply at 3 years, illustrating the poor diagnosis and the high rate of skeletal-related events (SREs), including pathological fractures, radiotherapy, surgery, neural compression, or hypercalcemia could reach 74–84% (27). However, the treatment aims at palliation that includes interferon-alpha interleukin-2 and targeted therapy with drugs based on tyrosine kinase inhibitors, TKIs, and mTOR inhibitors (29–31). We must intervene early in patients who are at high risk of KCBM and SREs to reduce the incidence of these disease and the occurrences of SREs. Historically, a nomogram was often used to establish a diagnosis evaluation model, but it has several limitations. There was no way to compensate, resulting in the removal of too many cases reducing the accuracy of the model. Through logistic analysis, traditional nomograms can only reached an AUC of 0.83 (16). A variety of machine learning algorithms and artificial intelligence systems have been developed as a result of advances in computer technology, and they are now being used more frequently in the field of medical biology to create diagnostic or prognosis models, offer solutions to automated decision support systems for personalized treatment, and perform other tasks that increase the effectiveness of the healthcare system (32).

We used descriptive statistics and logistic regression analysis to investigate variables related to KCBM at the time of diagnosis and exported the SHAP values to evaluate the impact of each factor. We found that younger people are more likely than elders to develop KCBM. Elderly patients are likely to have a worse prognosis. Evidence shows advanced age increases breast cancer's risk of bone metastasis. The fact that age is considered a protective factor for other cancers such as pancreatic cancer, may imply that age is type-specific as an independent risk factor for diagnosis (33, 34). Mitchell Fane and Ashani T. Weeraratna believe that age affects metastasis in several ways including changes in the immune microenvironment (inflaming, immunosenescenece, tumor-associated macrophages, myeloid-derived suppressor cells, regulatory T cell), that result in loss of tissue extracellular matrix integrity (35). Paradoxically, many factors involved in the evolution of age tissue that promote malignant transformation and hyperplastic growth contribute to the arrest of growth, apoptosis, and degradation of other cells and component of structural tissue components (36). We hypothesized that suppressive metastasis factors are stronger than prometastasis factors in KC's immune microenvironment and ECM of KC. Furthermore, most studies still agree that patients with KCBM will have a poorer prognosis of KCBM patients with aging (17, 37).

Treatment-wise, according to article written by George S. Karagiannis et al. on the residual breast cancer of patients treated with neoadjuvant paclitaxel after receiving doxorubicin plus cyclophosphamide, the density and activity of TMEM sites and Mena expression (a significant locus for tumor microenvironment of metastasis) increased. This suggests that chemotherapy, despite shrinking tumor size, increases the chance of metastatic (38). The effect of chemotherapy on BM in KC may be similar to that in breast cancer. In the SHAP graph, the characteristics of the factors confirmed that the application of chemotherapy plays a significant role in KCBM. In contrast, the prognosis SHAP graph illustrates the significance of chemotherapy treatment for the 5-year OS. Our study also depicted that the implementation of radiotherapy did not reduce BM rate and in KC patients or prolong survival time. International recommendations do not support the use of adjuvant radiation following nephrectomy. According to the Copenhagen Renal Cell Cancer Study Group's randomized experiment, radiation had no observable toxic effects and did not improve survival after 2 years (39). Nevertheless, to treat bone and brain metastases, radiotherapy, particularly stereotactic radiotherapy, can significantly relieve local systems (40). The mode, dose and mechanism of radiotherapy are complex. Radiotherapy's effects on controlling bone metastasis and prolong renal cancer prognosis depend on the mode and dose of delivery (41). In our study, surgery was deemed necessary to prevent metastasis and improve the prognosis.

SREs induced by KCBM can lead to reduce quality of life and an increase in health-care burdens (42). Hence, preventing SRE and KCBM is important for the management of patient with KC. In addition, the ESMO clinical practice guidelines suggest that treatment with anti-SRE drugs such as BMAs, denosumab is recommended for those who have a life expectancy >3 months (43). Therefore, accurate prediction of the prognosis and patients and identification of the predictive risk factors for BM are also important to guild the early initiation of anti-SREs treatment.

DT is commonly utilized for highly accurate tumor categorization and image screening (21, 44). A machine learning classifier called RF utilizes various trees to train and forecast variables that could reduce training variance and enhance integration and generalization (23, 45). MLP can use cross-entropy loss along with the stochastic gradient descent optimization with a momentum algorithm to improve the mode's performance (46). LR models are commonly used to validate the influence of trait variables on end events. LR models are seen as binary classifies (47). NBC is a model based on applying of the Bayes' theorem whose basic assumption is conditional independence of predictors based on the outcome (48). A machine learning algorithm called XGB that uses the gradient boosting framework (22, 49). SVM is often used to process gene expression profiles from tumor samples or peripheral blood for diagnosis or prognosis (20). Non-parametric classification methods like KMN are widely used; however, they can be impractical to implement with large databases because of memory consumption. Several techniques have been developed recently to improve these method (24). Using 10-fold cross-validation, optimal model hyperparameters were selected and fine-tuned by grid research. Overfitting was controlled by using the early-stop method (50).

Machine learning algorithms were suitable for observing associations between data beyond one-dimensional statistical methods such as logistic regression or Cox proportional hazard modeling. As computing power and storage space increase, machine learning algorithms can analyze more complex data and output instantaneously. Since traditional nomogram models must delete a large number of incomplete information cases, improving their prediction performance is always challenging. We maintained a large sample size, further enhanced by ten-fold cross-validation during model construction (51, 52). In recent years, XGB has become one of the most popular and innovative algorithms and has won the machine learning competition (49). Comparisons between our machine learning models with the TNM staging model and the other seven algorithms, including logistic regression, suggested that the XGB model incorporating clinical characteristics and treatment information input can effectively predict KCBM diagnosis and prognosis. A network calculator based on the XGB algorithm has been developed to visualize diagnostic and prognostic models and increase their speed and efficiency.

## Conclusion

To diagnose and prognosticate KCBM, we develop a variety of diagnostic and prognostic prediction models using machine learning and artificial intelligence technology. The XGB model was selected, and network tools were established after performance comparison. Using these models, clinicians can identify people individuals at high risk of BM and predict the prognostic of for patients with BM so that early treatment can improve prognosis and quality of life.

## Data availability statement

The original contributions presented in the study are included in the article/Supplementary material, further inquiries can be directed to the corresponding authors.

## Ethics statement

The studies involving human participants were reviewed and approved by Medical Ethics Committee of Zhejiang Provincial People's Hospital Zhejiang Provincial People's Hospital. Written informed consent for participation was not required for this study in accordance with the national legislation and the institutional requirements.

## Author contributions

QB and YK designed the project, reviewed, and edited the manuscript. LJ, WZ, and JH wrote the manuscript. JT and JL contributed to the literature retrieval. XZ and SZ carried out the research selection, data extraction, and statistical analysis. YT, ZH, and XM prepared the tables and figures. All authors contributed to this article and approved the submitted version.

## Funding

This study was supported by grants from National Science Foundation of China (Grant No. 81672769) and Major Science and Technology Projects of Zhejiang Province (2021C03078) as well as Medical Health Science and Technology Project of Zhejiang Provincial Health Commission, No. 2022ky583.

## Conflict of interest

The authors declare that the research was conducted in the absence of any commercial or financial relationships that could be construed as a potential conflict of interest.

## Publisher's note

All claims expressed in this article are solely those of the authors and do not necessarily represent those of their affiliated organizations, or those of the publisher, the editors and the reviewers. Any product that may be evaluated in this article, or claim that may be made by its manufacturer, is not guaranteed or endorsed by the publisher.

## Abbreviations

KCBM: kidney cancer bone metastasis
KC: kidney cancer
BM: bone metastasis
ccRCC: clear cell renal cell carcinom
SEER, Surveillance, Epidemiology: and End Results
AJCC: American Joint Committee on Cancer
ROC: receiver operating characteristic
DCA: Decision Curve Analysis
PR: Precision-Recall
LR: logistic regression
NBC: naive Bayes BS classifier
DT: decision tree
XGB: extreme gradient boosting
MLP: multilayer perceptron
RF: random forest
SVM: support vector machine
KNN: k-nearest neighbor
ICD-O: International Classification of Disease for Oncology
OS: overall survival
AUC: Area under the curve
TP: true positives
TN: true negatives
FP: false positives
FN: false negatives.
