# Supplementary material for: Bone metastasis risk and prognosis assessment models for kidney cancer based on machine learning
Source: Front Public Health. 2022 Nov 17;10:1015952. doi: 10.3389/fpubh.2022.1015952 (PMC9714267; doi:10.3389/fpubh.2022.1015952)
Supplement: Supplementary file 1 [file Data_Sheet_1.DOCX]

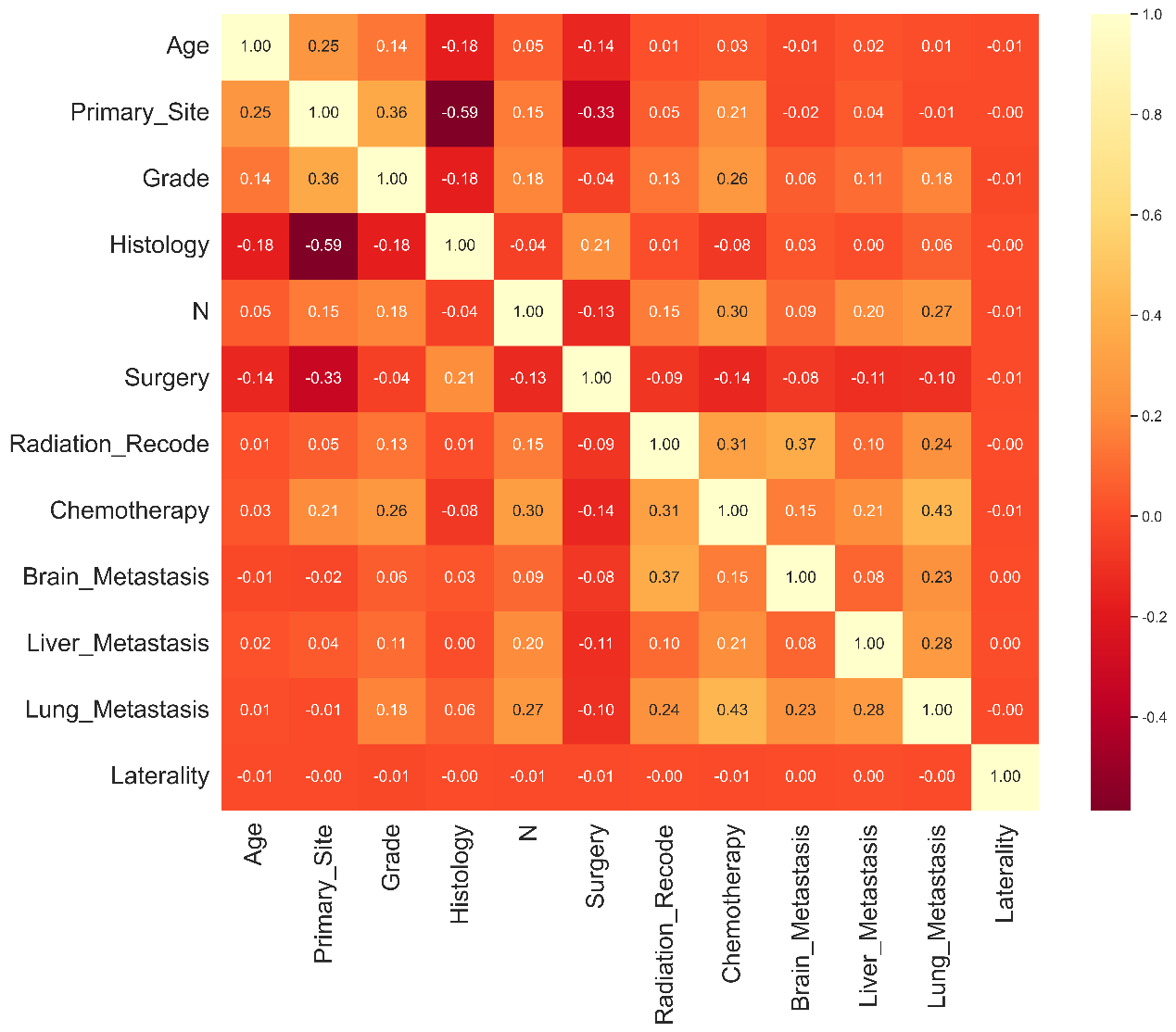


Figure S1. Results of correlation analysis between included variables of KCBM diagnosis model. The heat map shows the correlation between the variables.


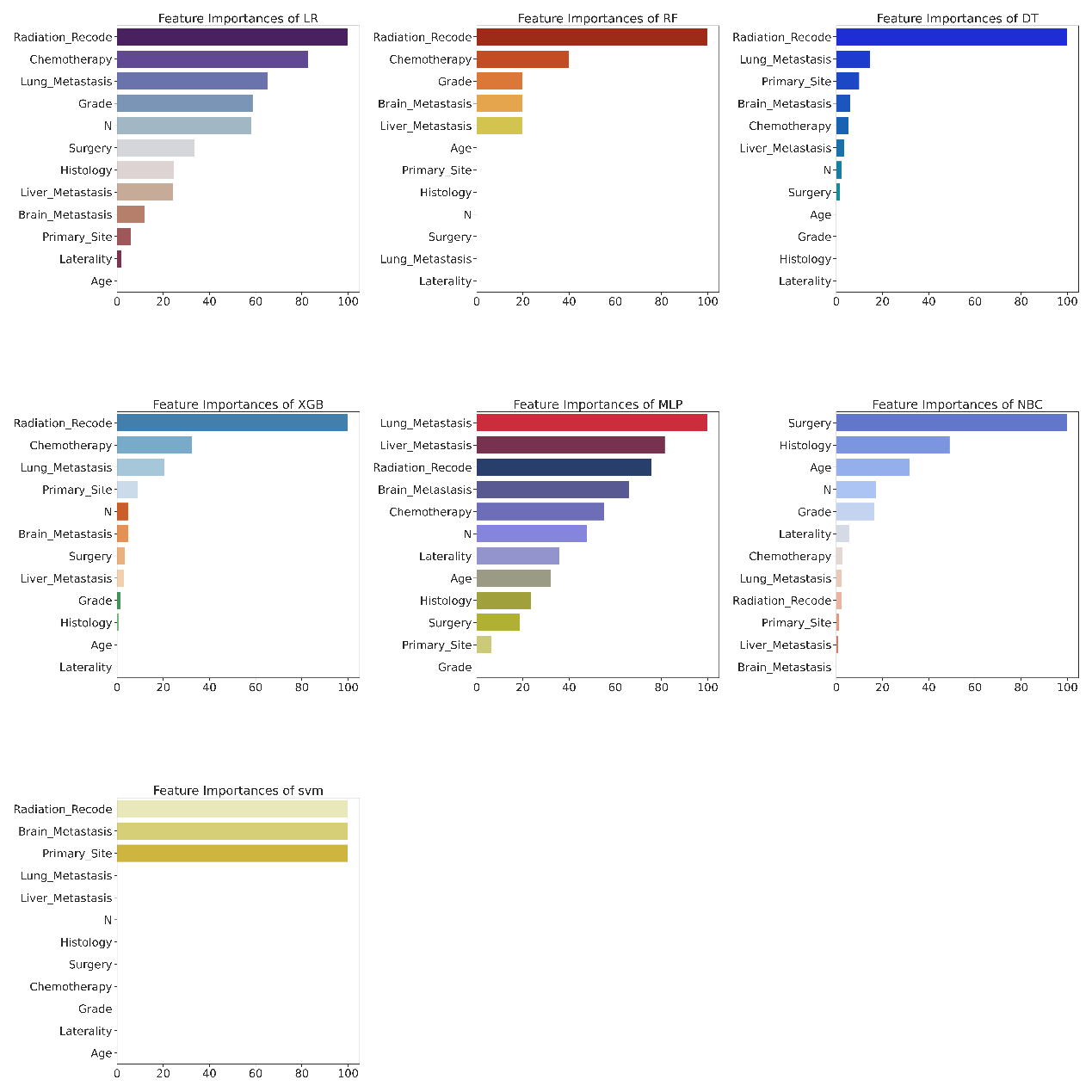


Figure S2. The feature importance in each machine learning model (diagnosis model).


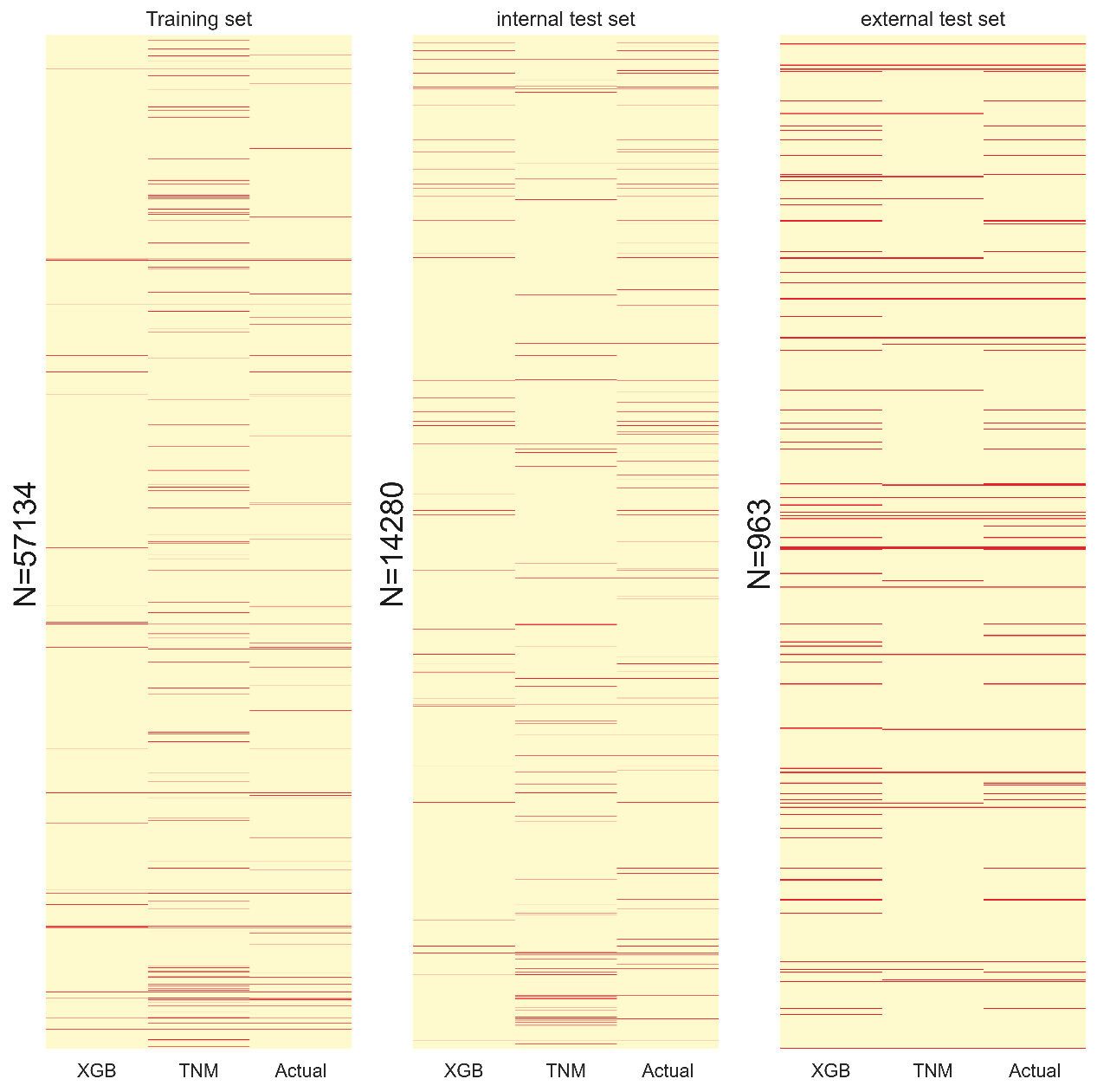


Figure S3. Prediction results of the XGB model and TNM staging model. The heat map shows the predicted results of XGB model versus the actual situation in internal test set and external test. Each column in the heat map represents the models’ predicted results of KCBM for all patients in the dataset. Dark colors represent KCBM cases and light colors are non-KCBM.

Figure S3. Results of correlation analysis between all variables of fracture model. The heat map shows the correlation between the variables


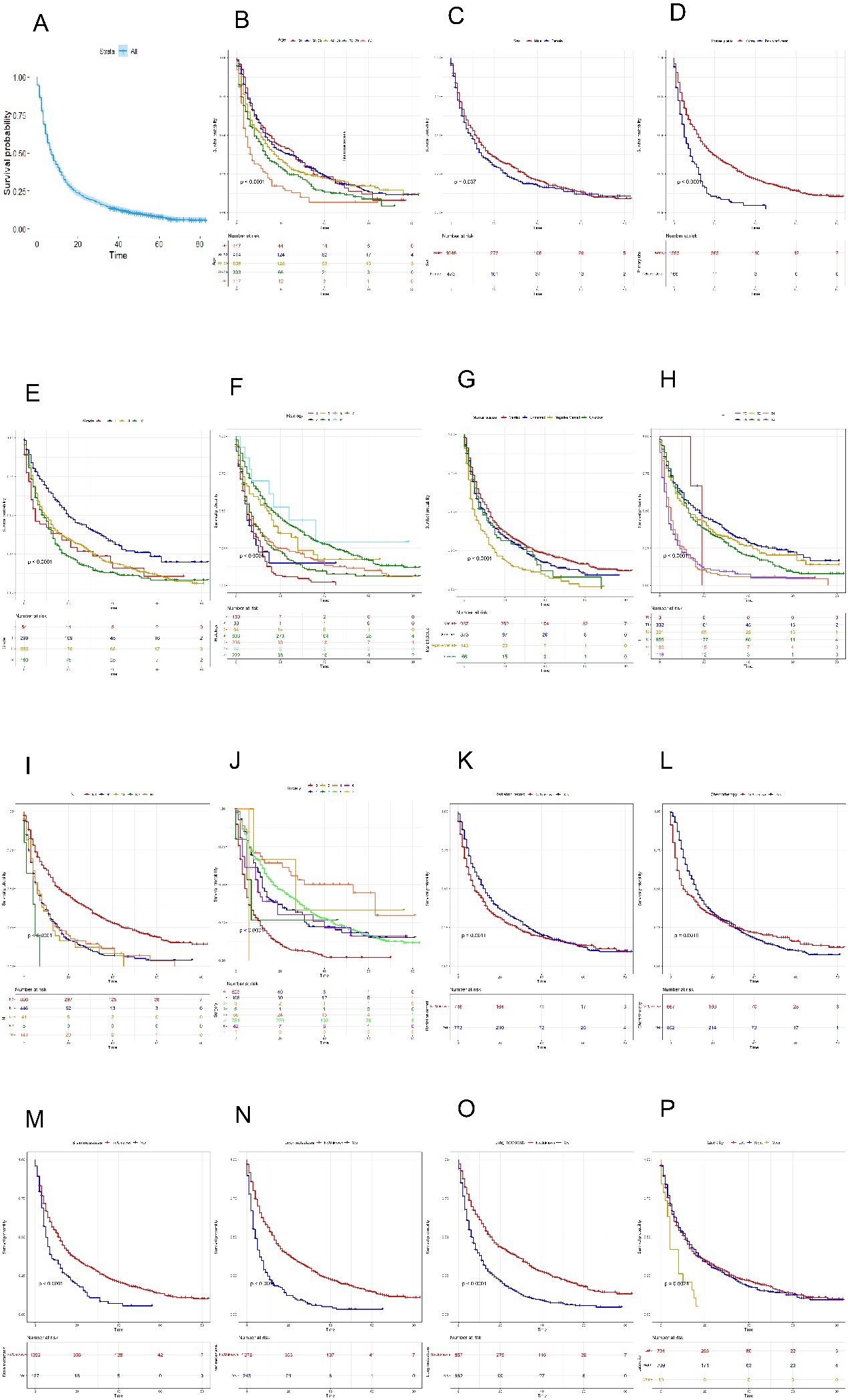


Figure S4. Kaplan-Meier analysis of overall survival among KCBM patients from the Surveillance, Epidemiology, and End Results (SEER) program database (2010–2016). A. overall. B. stratified by age. C. stratified by sex. D. stratified by primary site. E. stratified by grade. F. stratified by histology (1. transitional cell carcinoma 2. papillary transitional cell carcinoma 3. papillary adenocarcinoma 4. clear-cell adenocarcinoma 5. renal cell carcinoma 6. chromophobe type 7. other). G. stratified by marital status. H. stratified by T stage. I. stratified by N stage. J. stratified by surgery type (0. no surgery/unknown 1. complete/total/simple nephrectomy 2. local tumor destruction 3. local tumor excision 4. partial/subtotal nephrectomy/partial ureterectomy 5. radical nephrectomy 6. any nephrectomy 7. unknown) K. stratified by radiation record. L. stratified by chemotherapy. M. stratified by brain metastasis. N. stratified by liver metastasis. O. stratified by lung metastasis. p. stratified by laterality.


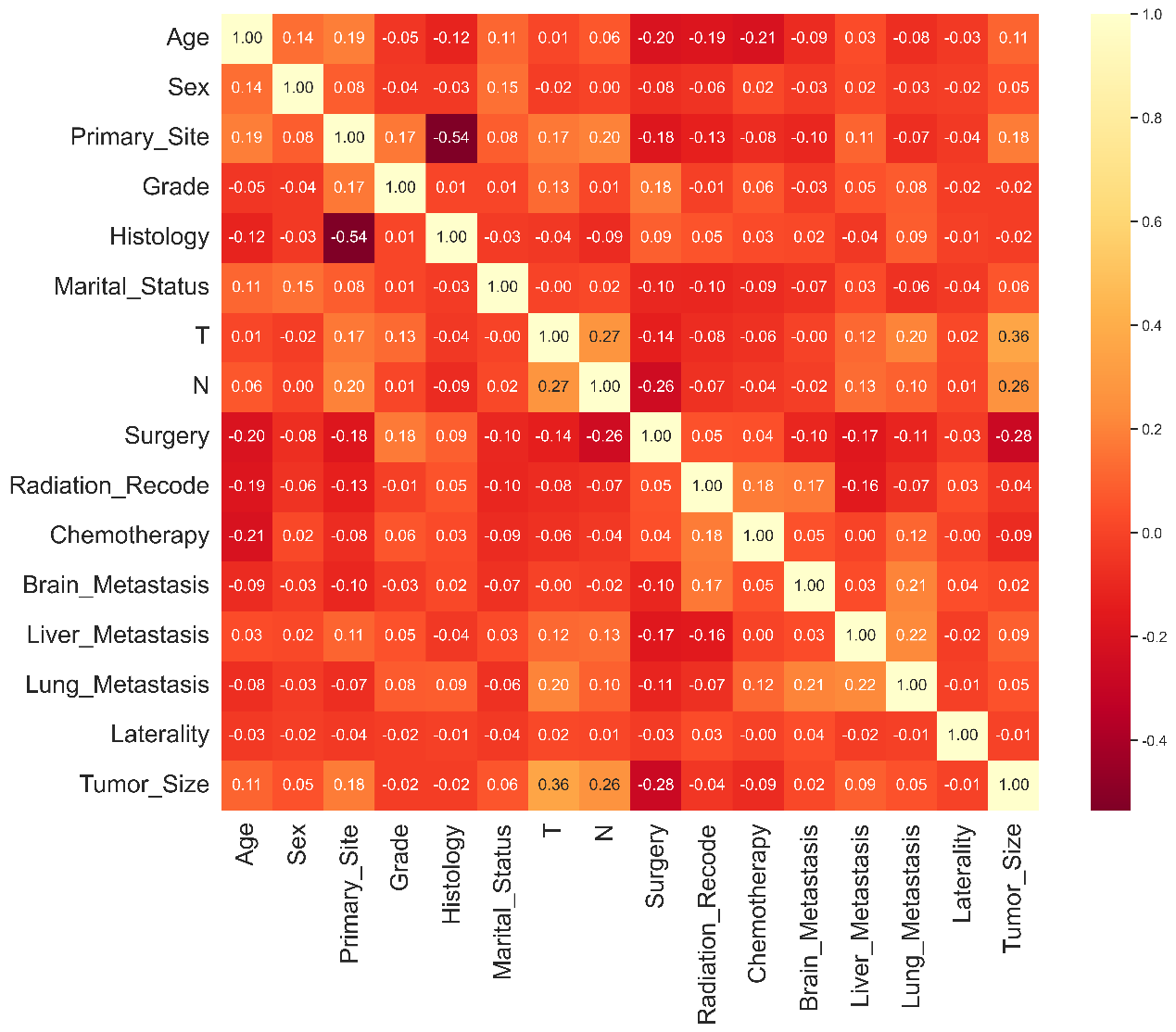


Figure S5. Results of correlation analysis between variables of prognosis model. The heat map shows the correlation between the variables.


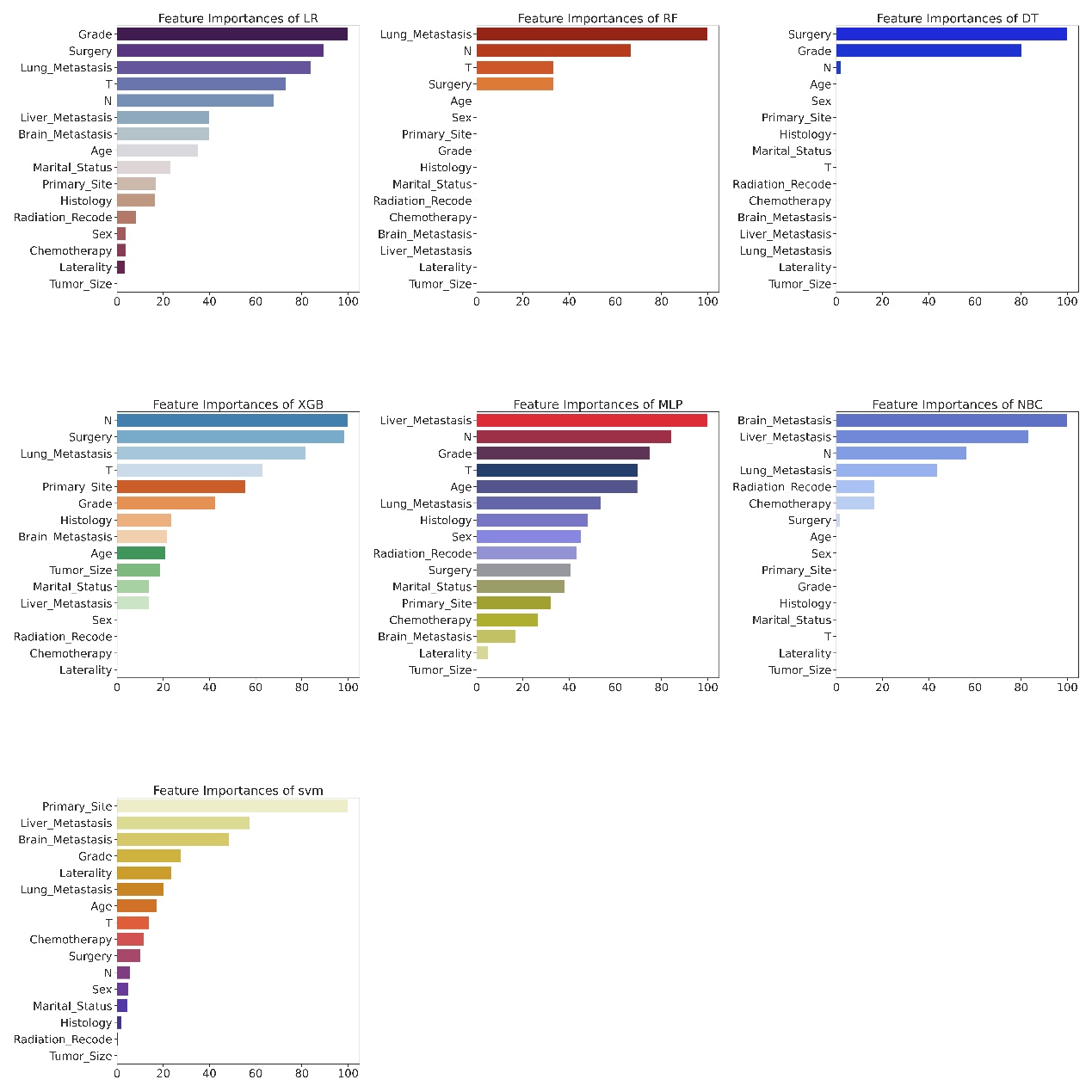


Figure 6. The feature importance in each machine learning model (prognosis model).


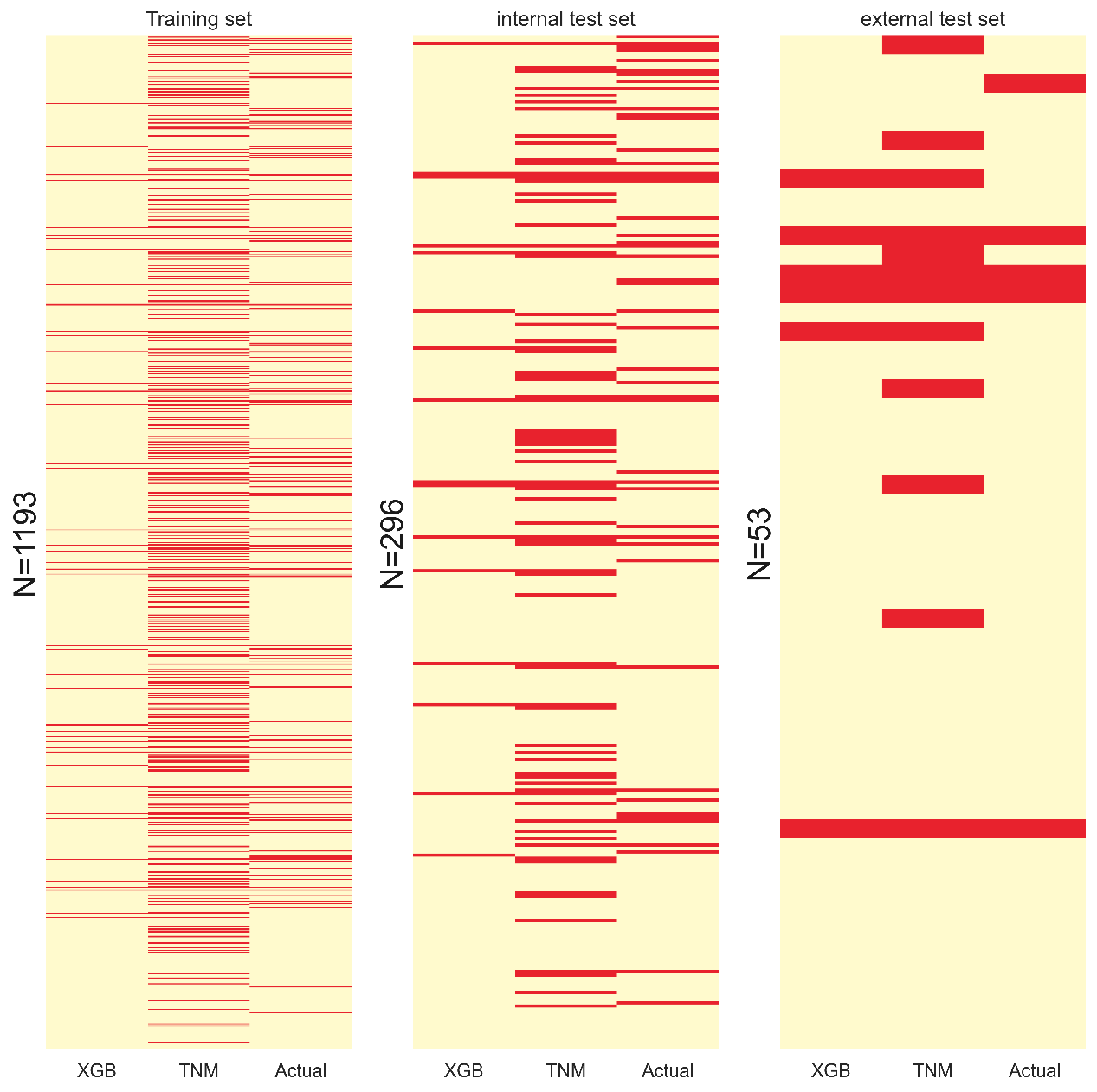


Figure S7. Prediction results of the XGB model and TNM staging moedel. The heat map shows the predicted results of XGB model versus the actual situation in internal test set and external test
