# Supplementary material for: Bone metastasis risk and prognosis assessment models for kidney cancer based on machine learning
Source: Front Public Health. 2022 Nov 17;10:1015952. doi: 10.3389/fpubh.2022.1015952 (PMC9714267; doi:10.3389/fpubh.2022.1015952)
Supplement: Supplementary file 2 [file Table_1.DOCX]

|  | **Training**  **group**  **(n=57134)** | **%** | **Validation**  **group**  **(n=14280)** | | **%** | **χ2** | **P** |
| --- | --- | --- | --- | --- | --- | --- | --- |
| **Age** |  |  |  |  | | 6.145 | 0.189 |
| <50 | 9011 | 15.7 | 2215 | 15.5 | |  |  |
| 50-59 | 13451 | 23.5 | 3330 | 23.3 | |  |  |
| 60-69 | 17778 | 31.1 | 4390 | 30.7 | |  |  |
| 70-79 | 12229 | 21.4 | 3098 | 21.6 | |  |  |
| >=80 | 4665 | 8.1 | 1247 | 8.7 | |  |  |
| **Race** |  |  |  |  | | 1.238 | 0.538 |
| White | 46911 | 82.1 | 11720 | 82.0 | |  |  |
| Black | 6502 | 11.3 | 1598 | 11.1 | |  |  |
| Other | 3721 | 6.5 | 962 | 6.7 | |  |  |
| **Sex** |  |  |  |  | | 1.739 | 0.187 |
| Male | 36408 | 63.7 | 9015 | 63.1 | |  |  |
| Female | 20726 | 36.2 | 5265 | 36.8 | |  |  |
| **Primary Site** |  |  |  |  | | 2.314 | 0.128 |
| Kidney | 51959 | 90.9 | 12928 | 90.5 | |  |  |
| Renal pelvis/Ureter | 5175 | 9.0 | 1352 | 9.4 | |  |  |
| **Grade** |  |  |  |  | | 3.074 | 0.380 |
| I | 6130 | 10.7 | 1560 | 10.9 | |  |  |
| II | 27094 | 47.4 | 6686 | 46.8 | |  |  |
| III | 16555 | 28.9 | 4122 | 28.8 | |  |  |
| IV | 7355 | 12.8 | 1902 | 13.3 | |  |  |
| **Histology** |  |  |  |  | | 4.845 | 0.564 |
| Transitional cell carcinoma | 2686za | 4.7 | 707 | 4.7 | |  |  |
| Papillary transitional cell carcinoma | 2709 | 4.7 | 713 | 4.9 | |  |  |
| Papillary adenocarcinoma | 6767 | 11.8 | 1697 | 11.9 | |  |  |
| Clear-cell adenocarcinoma | 34124 | 59.7 | 8507 | 59.5 | |  |  |
| Renal cell carcinoma | 5644 | 9.8 | 1388 | 9.7 | |  |  |
| Chromophobe type | 2157 | 3.7 | 535 | 5.7 | |  |  |
| Other | 3037 | 5.3 | 722 | 5.1 | |  |  |
| **Marital Status** |  |  |  |  | | 3.822 | 0.281 |
| Married | 34865 | 61.0 | 8692 | 60.8 | |  |  |
| Unmarried | 13915 | 24.3 | 3418 | 23.9 | |  |  |
| Negative married | 5442 | 9.5 | 1429 | 10.0 | |  |  |
| Unknown | 2912 | 5.0 | 741 | 5.1 | |  |  |
| **Insurance** |  |  |  |  | | 1.063 | 0.588 |
| Yes | 1398 | 2.4 | 329 | 2.3 | |  |  |
| No | 54944 | 96.1 | 13757 | 96.3 | |  |  |
| Unknown | 792 | 1.3 | 194 | 1.3 | |  |  |
| **Stage** |  |  |  |  | | 0.489 | 0.921 |
| I | 35838 | 62.7 | 8950 | 62.6 | |  |  |
| II | 5331 | 9.3 | 1312 | 9.1 | |  |  |
| III | 10186 | 17.8 | 2554 | 17.8 | |  |  |
| IV | 5779 | 10.1 | 1464 | 10.2 | |  |  |
| **T** |  |  |  |  | | 5.025 | 0.413 |
| T0 | 6 | <0.01 | 1 | <0.01 | |  |  |
| T1 | 35735 | 64.2 | 9163 | 64.1 | |  |  |
| T2 | 6142 | 10.7 | 1497 | 10.4 | |  |  |
| T3 | 12396 | 21.6 | 3137 | 21.9 | |  |  |
| T4 | 1496 | 2.6 | 375 | 2.6 | |  |  |
| TX | 359 | 0.6 | 107 | 0.7 | |  |  |
| **N** |  |  |  |  | | 2.399 | 0.663 |
| N0 | 53646 | 93.8 | 13403 | 93.8 | |  |  |
| N1 | 2526 | 4.4 | 654 | 4.5 | |  |  |
| N2 | 431x` | 0.7 | 93 | 0.6 | |  |  |
| N3 | 26 | <0.01 | 7 | <0.01 | |  |  |
| NX | 505 | 0.8 | 123 | 0.8 | |  |  |
| **M** |  |  |  |  | | 0.774 | 0.379 |
| M0 | 52545 | 91.9 | 13101 | 91.7 | |  |  |
| M1 | 4589 | 8.0 | 1179 | 8.2 | |  |  |
| **Surgery** |  |  |  |  | | 5.419 | 0.609 |
| No | 2570 | 4.4 | 667 | 4.7 | |  |  |
| Complete/total/simple nephrectomy | 6479 | 11.3 | 1629 | 11.4 | |  |  |
| Local tumor destruction | 1192 | 2.0 | 313 | 2.1 | |  |  |
| Local tumor excision | 987 | 1.7 | 243 | 1.7 | |  |  |
| Partial/subtotal nephrectomy/partial ureterectomy | 19414 | 33.9 | 4843 | 33.8 | |  |  |
| Radical nephrectomy | 25471 | 44.5 | 6300 | 44.1 | |  |  |
| Any nephrectomy | 1007 | 1.7 | 278 | 1.9 | |  |  |
| Unknown | 14 | <0.01 | 6 | <0.01 | |  |  |
| **Lymph Node Surgery** |  |  |  |  | | 2.894 | 0.235 |
| None/Unknown | 49404 | 86.4 | 12308 | 86.1 | |  |  |
| Lymph nodes removed | 7599 | 13.3 | 1929 | 13.5 | |  |  |
| Biopsy of lymph node | 131 | 0.2 | 43 | 0.3 | |  |  |
| **Radiation Therapy** |  |  |  |  | | 1.173 | 0.279 |
| None/Unknown | 55658 | 97.4 | 13888 | 97.2 | |  |  |
| Yes | 1476 | 2.5 | 392 | 2.7 | |  |  |
| **Chemotherapy** |  |  |  |  | | 0.005 | 0.943 |
| No/Unknown | 52835 | 92.4 | 13203 | 92.4 | |  |  |
| Yes | 4299 | 7.5 | 1077 | 7.5 | |  |  |
| **Brain Metastasis** |  |  |  |  | | 0.032 | 0.858 |
| No/Unknown | 56742 | 99.3 | 14184 | 99.3 | |  |  |
| Yes | 392 | 0.6 | 96 | 0.6 | |  |  |
| **Liver Metastasis** |  |  |  |  | | 2.374 | 0.123 |
| No/Unknown | 56349 | 98.6 | 14096 | 98.5 | |  |  |
| Yes | 785 | 1.3 | 221 | 1.4 | |  |  |
| **Lung Metastasis** |  |  |  |  | | 0.434 | 0.510 |
| No/Unknown | 54527 | 95.4 | 13610 | 95.3 | |  |  |
| Yes | 2607 | 4.5 | 670 | 4.6 | |  |  |
| **Laterality** |  |  |  |  | | 0.708 | 0.702 |
| Left | 28227 | 49.4 | 7018 | 49.1 | |  |  |
| Right | 28814 | 50.4 | 7242 | 50.7 | |  |  |
| Other | 93 | 0.1 | 20 | 0.1 | |  |  |

Table S1.Baseline clinical characteristics of KC patients.( We used t-test to verify the randomness of the tumor size, p=0.210 (95%CI, -4.375-0.960),)

|  | **Training Cohort** | | | | **Internal Validation Cohort** | | | **External Validation Cohort** | | |
| --- | --- | --- | --- | --- | --- | --- | --- | --- | --- | --- |
|  | **BM** | **No-BM** | **Total** |  | **BM** | **No-BM** | **Total** | **BM** | **No-BM** | **Total** |
| **Variable** | N=1491 | N=55643 | N=57134 | P | N=404 | N=13876 | N=179 | N=53 | N=910 | N=963 |
| **Age** |  |  |  | <0.001*** |  |  |  |  |  |  |
| <50 | 151 | 8860 | 9011 |  | 37 | 2178 | 2215 | 6 | 265 | 271 |
| 50-59 | 402 | 13049 | 13451 |  | 108 | 3222 | 3330 | 20 | 263 | 283 |
| 60-69 | 491 | 17287 | 17778 |  | 140 | 4250 | 4390 | 14 | 198 | 212 |
| 70-79 | 331 | 11898 | 12229 |  | 83 | 3015 | 3098 | 9 | 151 | 160 |
| >=80 | 116 | 4549 | 4665 |  | 36 | 1211 | 1247 | 4 | 33 | 37 |
| **Race** |  |  |  | 0.026* |  |  |  |  |  |  |
| White | 1253 | 45658 | 46911 |  | 344 | 11376 | 11720 | 0 | 0 | 0 |
| Black | 137 | 6365 | 6502 |  | 33 | 1565 | 1598 | 0 | 0 | 0 |
| Other | 101 | 3620 | 3721 |  | 27 | 935 | 962 | 53 | 910 | 963 |
| **Sex** |  |  |  | <0.001*** |  |  |  |  |  |  |
| Male | 1024 | 35384 | 36408 |  | 275 | 8740 | 9015 | 46 | 594 | 640 |
| Female | 467 | 20259 | 20726 |  | 129 | 5136 | 5265 | 7 | 316 | 323 |
| **Primary Site** |  |  |  | 0.001** |  |  |  |  |  |  |
| Kidney | 1321 | 50638 | 51959 |  | 363 | 12565 | 12928 | 44 | 752 | 796 |
| Renal pelvis/Ureter | 170 | 5005 | 5175 |  | 41 | 1311 | 1352 | 9 | 158 | 167 |
| **Grade** |  |  |  | <0.001*** |  |  |  |  |  |  |
| I | 55 | 6075 | 6130 |  | 14 | 1546 | 1560 | 3 | 468 | 471 |
| II | 298 | 26796 | 27094 |  | 70 | 6626 | 6696 | 13 | 331 | 344 |
| III | 655 | 15900 | 16555 |  | 186 | 3963 | 4149 | 14 | 74 | 88 |
| IV | 483 | 6872 | 7355 |  | 134 | 1769 | 1903 | 23 | 37 | 60 |
| **Histology** |  |  |  | <0.001*** |  |  |  |  |  |  |
| Transitional cell carcinoma | 150 | 2546 | 2696 |  | 35 | 672 | 707 | 7 | 78 | 85 |
| Papillary transitional cell carcinoma | 35 | 2674 | 2709 |  | 11 | 702 | 713 | 2 | 7 | 9 |
| Papillary adenocarcinoma | 62 | 6705 | 6767 |  | 14 | 1683 | 1697 | 14 | 59 | 73 |
| Clear-cell adenocarcinoma | 775 | 33349 | 34124 |  | 205 | 8302 | 8507 | 23 | 571 | 594 |
| Renal cell carcinoma | 235 | 5409 | 5644 |  | 73 | 1315 | 1388 | 9 | 103 | 112 |
| Chromophobe type | 11 | 2146 | 2157 |  | 6 | 529 | 535 | 1 | 19 | 20 |
| Other | 223 | 2814 | 3037 |  | 60 | 673 | 733 | 7 | 73 | 80 |
| **Marital Status** |  |  |  | 0.215 |  |  |  |  |  |  |
| Married | 920 | 33945 | 34865 |  | 245 | 8447 | 8692 | 52 | 856 | 908 |
| Unmarried | 374 | 13541 | 13915 |  | 98 | 3320 | 3418 | 1 | 22 | 23 |
| Negative married | 138 | 5304 | 5442 |  | 43 | 1386 | 1429 | 0 | 19 | 19 |
| Unknown | 59 | 2853 | 2912 |  | 18 | 723 | 741 | 0 | 13 | 13 |
| **Insurance** |  |  |  | 0.005** |  |  |  |  |  |  |
| No | 55 | 1343 | 1398 |  | 11 | 318 | 329 | 18 | 254 | 272 |
| Yes | 1412 | 53532 | 54944 |  | 389 | 13368 | 13757 | 35 | 595 | 630 |
| Unknown | 24 | 768 | 792 |  | 4 | 190 | 194 | 0 | 61 | 61 |
| **Stage** |  |  |  | <0.001*** |  |  |  |  |  |  |
| I | 0 | 35838 | 35838 |  | 0 | 8950 | 8950 | 0 | 632 | 632 |
| II | 0 | 5331 | 5331 |  | 0 | 1312 | 1312 | 0 | 144 | 144 |
| III | 1 | 10158 | 10159 |  | 0 | 2554 | 2554 | 0 | 113 | 113 |
| IV | 1490 | 4289 | 5779 |  | 404 | 1060 | 1464 | 53 | 21 | 74 |
| **T** |  |  |  | <0.001*** |  |  |  |  |  |  |
| T0 | 3 | 3 | 6 |  | 0 | 1 | 1 | 0 | 8 | 8 |
| T1 | 310 | 36425 | 36735 |  | 81 | 9082 | 9163 | 15 | 646 | 661 |
| T2 | 222 | 5920 | 6142 |  | 53 | 1444 | 1497 | 12 | 152 | 164 |
| T3 | 639 | 11757 | 12396 |  | 166 | 2971 | 3137 | 9 | 92 | 101 |
| T4 | 183 | 1313 | 1496 |  | 54 | 321 | 375 | 17 | 11 | 28 |
| TX | 134 | 225 | 359 |  | 9 | 57 | 66 | 0 | 1 | 1 |
| **N** |  |  |  |  |  |  |  |  |  |  |
| N0 | 894 | 52752 | 53646 |  | 221 | 13182 | 13403 | 18 | 862 | 880 |
| N1 | 425 | 2101 | 2526 | <0.001*** | 126 | 528 | 654 | 2 | 40 | 42 |
| N2 | 47 | 384 | 431 |  | 10 | 83 | 93 | 4 | 1 | 5 |
| N3 | 4 | 22 | 26 |  | 2 | 5 | 7 | 9 | 6 | 15 |
| NX | 121 | 384 | 505 |  | 45 | 78 | 123 | 0 | 1 | 1 |
| **M** |  |  |  | <0.001*** |  |  |  |  |  |  |
| M0 | 0 | 52544 | 52544 |  | 0 | 13101 | 13101 | 0 | 892 | 892 |
| M1 | 1491 | 3099 | 4590 |  | 404 | 775 | 1179 | 53 | 18 | 71 |
| **Surgery** |  |  |  | <0.001*** |  |  |  |  |  |  |
| No | 514 | 2056 | 2570 |  | 148 | 529 | 677 | 13 | 19 | 32 |
| Complete/total/simple nephrectomy | 107 | 6372 | 6479 |  | 30 | 1599 | 1629 | 1 | 17 | 18 |
| Local tumor destruction | 3 | 1189 | 1192 |  | 1 | 312 | 313 | 1 | 2 | 3 |
| Local tumor excision | 7 | 980 | 987 |  | 2 | 241 | 243 | 6 | 63 | 69 |
| Partial/subtotal nephrectomy/partial ureterectomy | 59 | 19355 | 19414 |  | 14 | 4820 | 4834 | 1 | 439 | 440 |
| Radical nephrectomy | 761 | 24710 | 25471 |  | 194 | 6160 | 6354 | 31 | 265 | 296 |
| Any nephrectomy | 39 | 968 | 1007 |  | 15 | 263 | 278 | 0 | 0 | 0 |
| Unknown | 1 | 13 | 14 |  | 0 | 6 | 6 | 0 | 5 | 5 |
| **Lymph Node Surgery** |  |  |  | <0.001*** |  |  |  |  |  |  |
| None/Unknown | 1157 | 48247 | 49404 |  | 302 | 12006 | 12308 | 43 | 859 | 902 |
| Lymph nodes removed | 325 | 7274 | 7599 |  | 95 | 1834 | 1929 | 10 | 46 | 56 |
| Biopsy of lymph node | 9 | 122 | 131 |  | 7 | 36 | 43 | 0 | 5 | 5 |
| **Radiation Therapy** |  |  |  | <0.001*** |  |  |  |  |  |  |
| None/Unknown | 730 | 54928 | 55658 |  | 192 | 13696 | 13888 | 43 | 906 | 949 |
| Yes | 761 | 715 | 1476 |  | 212 | 180 | 392 | 11 | 4 | 15 |
| **Chemotherapy** |  |  |  | <0.001*** |  |  |  |  |  |  |
| No/Unknown | 632 | 52203 | 52835 |  | 187 | 13016 | 13203 | 26 | 873 | 899 |
| Yes | 859 | 3440 | 4299 |  | 217 | 860 | 1077 | 27 | 37 | 64 |
| **Brain Metastasis** |  |  |  | <0.001*** |  |  |  |  |  |  |
| No/Unknown | 1361 | 55381 | 56742 |  | 317 | 13813 | 14130 | 48 | 910 | 958 |
| Yes | 130 | 262 | 392 |  | 33 | 63 | 96 | 5 | 0 | 5 |
| **Liver Metastasis** |  |  |  | <0.001*** |  |  |  |  |  |  |
| No/Unknown | 1253 | 55096 | 56349 |  | 339 | 13730 | 14069 | 44 | 905 | 949 |
| Yes | 238 | 547 | 785 |  | 65 | 146 | 211 | 9 | 5 | 14 |
| **Lung Metastasis** |  |  |  | <0.001*** |  |  |  |  |  |  |
| No/Unknown | 836 | 53691 | 54527 |  | 230 | 13380 | 13610 | 33 | 899 | 932 |
| Yes | 655 | 1952 | 2607 |  | 174 | 496 | 670 | 20 | 11 | 31 |
| **Laterality** |  |  |  | <0.001*** |  |  |  |  |  |  |
| Left | 775 | 27452 | 28227 |  | 205 | 6813 | 7018 | 32 | 456 | 488 |
| Right | 700 | 28114 | 28814 |  | 194 | 7048 | 7242 | 19 | 442 | 461 |
| Other | 16 | 77 | 93 |  | 5 | 15 | 20 | 2 | 12 | 14 |

Table S2. Distribution of variables according to bone metastasis status

| **Factors** | **Univariate Analysis** | **Multivariate Logistic Analysis** | | |
| --- | --- | --- | --- | --- |
|  | **P** | **OR** | **95%CI** | **P** |
| **Age** | <0.001*** |  |  |  |
| <50 |  | - | - | - |
| 50-59 |  | 1.535 | 1.207-1.961 | <0.001*** |
| 60-69 |  | 1.437 | 1.135-1.828 | 0.002** |
| 70-79 |  | 1.445 | 1.121-1.870 | 0.004** |
| >=80 |  | 1.292 | 0.933-1.786 | 0.120 |
| **Race** | <0.001*** |  |  |  |
| White |  | - | - | - |
| Black |  | 1.063 | 0.839-1.337 | 0.602 |
| Other |  | 0.961 | 0.739-1.236 | 0.763 |
| **Sex** | <0.001*** |  |  |  |
| Male |  | - | - | - |
| Female |  | 0.959 | 0.830-1.108 | 0.577 |
| **Primary Site** | 0.001** |  |  |  |
| Kidney |  | - | - | - |
| Renal pelvis/Ureter |  | 0.477 | 0.314-0.726 | <0.001*** |
| **Grade** | <0.001*** |  |  |  |
| I |  | - | - | - |
| II |  | 1.144 | 0.821-1.623 | 0.436 |
| III |  | 2.067 | 1.493-2.917 | <0.001*** |
| IV |  | 2.063 | 1.456-2.970 | <0.001*** |
| **Histology** | <0.001*** |  |  |  |
| Transitional cell carcinoma |  | - | - | - |
| Papillary transitional cell carcinoma |  | 0.685 | 0.437-1.048 | 0.089 |
| Papillary adenocarcinoma |  | 0.692 | 0.423-1.133 | 0.144 |
| Clear-cell adenocarcinoma |  | 0.994 | 0.664-1.500 | 0.977 |
| Renal cell carcinoma |  | 1.009 | 0.660-1.555 | 0.963 |
| Chromophobe type |  | 0.451 | 0.199-0.939 | 0.042* |
| Other |  | 1.125 | 0.751-1.693 | 0.567 |
| **Marital Status** | 0.046* |  |  |  |
| Married |  | - | - | - |
| Unmarried |  | 1.081 | 0.920-1.268 | 0.336 |
| Negative married |  | 1.109 | 0.870-1.404 | 0.394 |
| Unknown |  | 1.094 | 0.778-1.510 | 0.591 |
| **Insurance** | 0.001* |  |  |  |
| Yes |  | - | - | - |
| No |  | 0.877 | 0.614-1.280 | 0.486 |
| Unknown |  | 1.551 | 0.826-2.840 | 0.162 |
| **Stage** |  |  |  |  |
| I | - |  |  |  |
| II | 1.000 |  |  |  |
| III | 0.936 |  |  |  |
| IV | 0.894 |  |  |  |
| **T** | <0.001*** |  |  |  |
| T0 |  | - | - | - |
| T1 |  | 0.282 | 0.032-2.425 | 0.239 |
| T2 |  | 0.411 | 0.046-3.543 | 0.409 |
| T3 |  | 0.380 | 0.043-3.263 | 0.368 |
| T4 |  | 0.340 | 0.038-2.935 | 0.317 |
| TX |  | 0.853 | 0.097-7.349 | 0.883 |
| **N** | <0.001*** |  |  |  |
| N0 |  | - | - | - |
| N1 |  | 1.824 | 1.512-2.199 | 0.883 |
| N2 |  | 2.202 | 1.396-3.432 | <0.001*** |
| N3 |  | 1.295 | 0.265-5.357 | <0.001*** |
| NX |  | 2.081 | 1.517-2.837 | 0.734 |
| **Surgery** | <0.001*** |  |  |  |
| No |  | - | - | - |
| Complete/total/simple nephrectomy |  | 0.264 | 0.195-0.356 | <0.001*** |
| Local tumor destruction |  | 0.117 | 0.028-0.323 | <0.001*** |
| Local tumor excision |  | 0.162 | 0.062-0.062 | <0.001*** |
| Partial/subtotal nephrectomy/partial ureterectomy |  | 0.124 | 0.088-0.172 | <0.001*** |
| Radical nephrectomy |  | 0.414 | 0.336-0.511 | <0.001*** |
| Any nephrectomy |  | 0.407 | 0.261-0.618 | <0.001*** |
| Unknown |  | 5.052 | 0.274-26.322 | 0.123 |
| **Lymph Node Surgery** | <0.001*** |  |  |  |
| None/Unknown |  | - | - | - |
| Lymph nodes removed |  | 0.834 | 0.690-1.004 | 0.057 |
| Biopsy of lymph node |  | 0.598 | 0.242-1.315 | 0.230 |
| **Radiation Therapy** | <0.001*** |  |  |  |
| None/Unknown |  | - | - | - |
| Yes |  | 35.253 | 29.978-41.508 | <0.001*** |
| **Chemotherapy** | <0.001*** |  |  |  |
| No/Unknown |  | - | - | - |
| Yes |  | 2.926 | 2.507-3.415 | <0.001*** |
| **Brain Metastasis** | <0.001*** |  |  |  |
| No/Unknown |  | - | - | - |
| Yes |  | 0.236 | 0.174-0.319 | <0.001*** |
| **Liver Metastasis** | <0.001*** |  |  |  |
| No/Unknown |  | - | - | - |
| Yes |  | 2.676 | 2.133-3.349 | <0.001*** |
| **Lung Metastasis** | <0.001*** |  |  |  |
| No/Unknown |  | - | - | - |
| Yes |  | 2.684 | 2.266-3.178 | <0.001*** |
| **Laterality** | <0.001*** |  |  |  |
| Left |  | - | - | - |
| Right |  | 0.887 | 0.777-1.013 | 0.078 |
| Other |  | 0.415 | 0.170-0.957 | 0.045 |
| **Tumor Size** | <0.001*** | 1.000 | 0.999-1.000 | 0.683 |

Table S3. Univariate and multivariate logistic analyses of KCBM

|  | **Training**  **group**  **(n=1193)** | **%** | **Validation**  **group**  **(n=296)** | | **%** | **χ2** | **P** |
| --- | --- | --- | --- | --- | --- | --- | --- |
| **Age** |  |  |  |  | | 0.154 | 0.695 |
| <50 | 122 | 10.8 | 15 | 5.0 | |  |  |
| 50-59 | 299 | 25.0 | 92 | 31.0 | |  |  |
| 60-69 | 396 | 33.1 | 100 | 33.7 | |  |  |
| 70-79 | 261 | 21.8 | 68 | 22.9 | |  |  |
| >=80 | 108 | 9.0 | 21 | 7.0 | |  |  |
| **Race** |  |  |  |  | | 0.109 | 0.947 |
| White | 1001 | 83.9 | 248 | 83.7 | |  |  |
| Black | 107 | 8.9 | 28 | 9.4 | |  |  |
| Other | 85 | 9.1 | 20 | 6.7 | |  |  |
| **Sex** |  |  |  |  | | 0.037 | 0.848 |
| Male | 817 | 68.4 | 201 | 67.9 | |  |  |
| Female | 376 | 31.5 | 95 | 32.0 | |  |  |
| **Primary Site** |  |  |  |  | | 8.160 | 0.004 |
| Kidney | 1056 | 88.5 | 252 | 85.1 | |  |  |
| Renal pelvis/Ureter | 107 | 11.4 | 44 | 14.1 | |  |  |
| **Grade** |  |  |  |  | | 3.460 | 0.326 |
| I | 47 | 3.9 | 11 | 3.7 | |  |  |
| II | 204 | 17.0 | 61 | 20.6 | |  |  |
| III | 554 | 46.7 | 122 | 41.2 | |  |  |
| IV | 384 | 32.1 | 102 | 34.3 | |  |  |
| **Histology** |  |  |  |  | | 4.379 | 0.626 |
| Transitional cell carcinoma | 121 | 10.1 | 40 | 13.5 | |  |  |
| Papillary transitional cell carcinoma | 30 | 2.5 | 7 | 2.3 | |  |  |
| Papillary adenocarcinoma | 48 | 4.0 | 10 | 3.3 | |  |  |
| Clear-cell adenocarcinoma | 573 | 48.0 | 147 | 49.6 | |  |  |
| Renal cell carcinoma | 212 | 17.7 | 47 | 15.8 | |  |  |
| Chromophobe type | 10 | 0.8 | 3 | 1.0 | |  |  |
| Other | 199 | 16.6 | 42 | 14.1 | |  |  |
| **Marital Status** |  |  |  |  | | 2.144 | 0.543 |
| Married | 733 | 61.4 | 170 | 57.4 | |  |  |
| Unmarried | 288 | 24.1 | 78 | 26.3 | |  |  |
| Negative married | 127 | 10.6 | 33 | 11.1 | |  |  |
| Unknown | 45 | 3.7 | 15 | 5.0 | |  |  |
| **Insurance** |  |  |  |  | | 0.962 | 0.618 |
| Yes | 43 | 3.6 | 8 | 2.7 | |  |  |
| No | 1132 | 94.8 | 282 | 95.2 | |  |  |
| Unknown | 18 | 1.5 | 6 | 2.0 | |  |  |
| **T** |  |  |  |  | | 5.607 | 0.346 |
| T0 | 1 | <0.01 | 1 | 0.3 | |  |  |
| T1 | 240 | 20.1 | 55 | 18.5 | |  |  |
| T2 | 175 | 14.6 | 36 | 12.1 | |  |  |
| T3 | 478 | 40.0 | 128 | 43.2 | |  |  |
| T4 | 177 | 14.8 | 37 | 12.5 | |  |  |
| TX | 122 | 10.2 | 39 | 13.1 | |  |  |
| **N** |  |  |  |  | | 4.563 | 0.335 |
| N0 | 673 | 93.8 | 158 | 53.7 | |  |  |
| N1 | 375 | 4.4 | 89 | 30.0 | |  |  |
| N2 | 39 | 0.7 | 10 | 3.3 | |  |  |
| N3 | 3 | <0.01 | 2 | 0.6 | |  |  |
| NX | 103 | 0.8 | 36 | 12.1 | |  |  |
| **Surgery** |  |  |  |  | | 7.863 | 0.345 |
| No | 457 | 38.5 | 117 | 39.5 | |  |  |
| Complete/total/simple nephrectomy | 86 | 7.2 | 24 | 8.0 | |  |  |
| Local tumor destruction | 3 | 0.2 | 0 | 0 | |  |  |
| Local tumor excision | 6 | 0.5 | 1 | 0.3 | |  |  |
| Partial/subtotal nephrectomy/partial ureterectomy | 44 | 3.6 | 6 | 2.0 | |  |  |
| Radical nephrectomy | 565 | 47.3 | 134 | 45.2 | |  |  |
| Any nephrectomy | 31 | 2.5 | 14 | 4.7 | |  |  |
| Unknown | 1 | <0.01 | 0 | 0 | |  |  |
| **Lymph Node Surgery** |  |  |  |  | | 5.039 | 0.081 |
| None/Unknown | 944 | 79.1 | 216 | 72.9 | |  |  |
| Lymph nodes removed | 240 | 20.1 | 77 | 26.0 | |  |  |
| Biopsy of lymph node | 9 | 0.7 | 3 | 1.0 | |  |  |
| **Radiation Therapy** |  |  |  |  | | 0.161 | 0.688 |
| None/Unknown | 589 | 49.3 | 150 | 50.6 | |  |  |
| Yes | 604 | 50.6 | 146 | 49.3 | |  |  |
| **Chemotherapy** |  |  |  |  | | 0.044 | 0.834 |
| No/Unknown | 532 | 44.5 | 134 | 45.2 | |  |  |
| Yes | 661 | 55.4 | 162 | 54.7 | |  |  |
| **Brain Metastasis** |  |  |  |  | | 3.171 | 0.075 |
| No/Unknown | 1072 | 89.8 | 276 | 93.2 | |  |  |
| Yes | 121 | 10.1 | 20 | 6.7 | |  |  |
| **Liver Metastasis** |  |  |  |  | | 0.441 | 0.507 |
| No/Unknown | 983 | 82.8 | 239 | 80.7 | |  |  |
| Yes | 210 | 17.6 | 57 | 19.2 | |  |  |
| **Lung Metastasis** |  |  |  |  | | 0.363 | 0.547 |
| No/Unknown | 652 | 54.6 | 156 | 53.7 | |  |  |
| Yes | 541 | 45.3 | 140 | 47.2 | |  |  |
| **Laterality** |  |  |  |  | | 3.303 | 0.192 |
| Left | 632 | 52.9 | 142 | 47.9 | |  |  |
| Right | 547 | 45.8 | 148 | 50.0 | |  |  |
| Other | 14 | 1.1 | 6 | 2.0 | |  |  |

Table S4. Baseline clinical characteristics of KCBM patients.( We used t-test to verify the randomness of the tumor size, p=0.108 (95%CI, -63.558-6.248),)

|  | **Training Cohort** | | | | **Internal Validation Cohort** | | | **External Validation Cohort** | | |
| --- | --- | --- | --- | --- | --- | --- | --- | --- | --- | --- |
|  | **Alive** | **Dead** | **Total** |  | **Alive** | **Dead** | **Total** | **Alive** | **Dead** | **Total** |
| **Variable** | N=100 | N=1093 | N=1193 | P | N=31 | N=265 | N=296 | N=3 | N=50 | N=53 |
| **Age** |  |  |  | 0.118 |  |  |  |  |  |  |
| <50 | 9 | 120 | 129 |  | 0 | 15 | 15 | 0 | 6 | 6 |
| 50-59 | 29 | 270 | 299 |  | 13 | 79 | 92 | 1 | 19 | 20 |
| 60-69 | 40 | 356 | 396 |  | 15 | 85 | 100 | 2 | 12 | 14 |
| 70-79 | 19 | 242 | 261 |  | 2 | 66 | 68 | 0 | 9 | 9 |
| >=80 | 3 | 105 | 108 |  | 1 | 20 | 21 | 0 | 4 | 4 |
| **Race** |  |  |  | 0.767 |  |  |  |  |  |  |
| White | 86 | 915 | 1001 |  | 26 | 222 | 248 | 0 | 0 | 0 |
| Black | 7 | 100 | 107 |  | 4 | 24 | 28 | 0 | 0 | 0 |
| Other | 7 | 78 | 85 |  | 1 | 19 | 20 | 3 | 50 | 53 |
| **Sex** |  |  |  | 0.571 |  |  |  |  |  |  |
| Male | 71 | 746 | 817 |  | 26 | 175 | 201 | 3 | 43 | 46 |
| Female | 29 | 347 | 376 |  | 5 | 90 | 95 | 0 | 7 | 7 |
| **Primary Site** |  |  |  | 0.001** |  |  |  |  |  | 0 |
| Kidney | 99 | 957 | 1056 |  | 29 | 223 | 252 | 3 | 41 | 44 |
| Renal pelvis/Ureter | 1 | 136 | 137 |  | 2 | 42 | 44 | 0 | 9 | 9 |
| **Grade** |  |  |  | <0.001*** |  |  |  |  |  |  |
| I | 6 | 41 | 47 |  | 0 | 11 | 11 | 0 | 3 | 3 |
| II | 32 | 172 | 204 |  | 14 | 47 | 61 | 2 | 11 | 13 |
| III | 46 | 512 | 558 |  | 9 | 113 | 122 | 0 | 14 | 14 |
| IV | 16 | 368 | 384 |  | 8 | 94 | 102 | 1 | 22 | 23 |
| **Histology** |  |  | 0 | <0.001*** |  |  |  |  |  |  |
| Transitional cell carcinoma | 0 | 121 | 121 |  | 2 | 38 | 40 | 0 | 7 | 7 |
| Papillary transitional cell carcinoma | 1 | 29 | 30 |  | 2 | 7 | 9 | 0 | 2 | 2 |
| Papillary adenocarcinoma | 4 | 44 | 48 |  | 20 | 8 | 28 | 0 | 4 | 4 |
| Clear-cell adenocarcinoma | 75 | 498 | 573 |  | 4 | 127 | 131 | 2 | 21 | 23 |
| Renal cell carcinoma | 12 | 100 | 112 |  | 1 | 43 | 44 | 0 | 9 | 9 |
| Chromophobe type | 1 | 9 | 10 |  | 2 | 2 | 4 | 0 | 1 | 1 |
| Other | 7 | 292 | 299 |  | 0 | 40 | 40 | 1 | 6 | 7 |
| **Marital Status** |  |  |  | 0.008** |  |  |  |  |  |  |
| Married | 76 | 657 | 733 |  | 18 | 152 | 170 | 3 | 49 | 52 |
| Unmarried | 19 | 269 | 288 |  | 11 | 67 | 78 | 0 | 0 | 0 |
| Negative married | 3 | 124 | 127 |  | 1 | 32 | 33 | 0 | 1 | 1 |
| Unknown | 2 | 43 | 45 |  | 1 | 14 | 15 | 0 | 0 | 0 |
| **Insurance** |  |  |  | 0.867 |  |  |  |  |  |  |
| No | 3 | 40 | 43 |  | 2 | 6 | 0 | 0 | 18 | 18 |
| Yes | 95 | 1037 | 1132 |  | 29 | 253 | 2 | 3 | 32 | 35 |
| Unknown | 2 | 16 | 18 |  | 0 | 6 | 0 | 0 | 0 | 0 |
| **T** |  |  |  | 0.045* |  |  |  |  |  |  |
| T0 | 3 | 1 | 4 |  | 0 | 1 | 1 | 0 | 0 | 0 |
| T1 | 310 | 201 | 511 |  | 12 | 43 | 55 | 2 | 13 | 15 |
| T2 | 222 | 155 | 377 |  | 6 | 30 | 36 | 1 | 11 | 12 |
| T3 | 639 | 444 | 1083 |  | 11 | 117 | 128 | 0 | 9 | 9 |
| T4 | 183 | 172 | 355 |  | 2 | 35 | 37 | 0 | 17 | 17 |
| TX | 134 | 120 | 254 |  | 0 | 39 | 39 | 0 | 0 | 0 |
| **N** |  |  |  | 0.023* |  |  |  |  |  |  |
| N0 | 894 | 583 | 1477 |  | 0 | 137 | 137 | 3 | 15 | 18 |
| N1 | 425 | 366 | 791 |  | 31 | 85 | 116 | 0 | 22 | 22 |
| N2 | 47 | 39 | 86 |  | 0 | 9 | 9 | 0 | 0 | 0 |
| N3 | 4 | 3 | 7 |  | 0 | 2 | 2 | 0 | 4 | 4 |
| NX | 121 | 102 | 223 |  | 0 | 32 | 32 | 0 | 0 | 0 |
| **Surgery** |  |  |  | 0.028* |  |  |  |  |  | 0 |
| No | 514 | 454 | 968 |  | 5 | 112 | 117 | 0 | 13 | 13 |
| Complete/total/simple nephrectomy | 107 | 80 | 187 |  | 3 | 21 | 24 | 0 | 1 | 1 |
| Local tumor destruction | 3 | 2 | 5 |  | 0 | 0 | 0 | 0 | 1 | 1 |
| Local tumor excision | 7 | 5 | 12 |  | 0 | 1 | 1 | 1 | 6 | 7 |
| Partial/subtotal nephrectomy/partial ureterectomy | 59 | 30 | 89 |  | 3 | 3 | 6 | 2 | 0 | 2 |
| Radical nephrectomy | 761 | 494 | 1255 |  | 19 | 115 | 134 | 0 | 29 | 29 |
| Any nephrectomy | 39 | 27 | 66 |  | 1 | 13 | 14 | 0 | 0 | 0 |
| Unknown | 1 | 1 | 2 |  | 0 | 0 | 0 | 0 | 0 | 0 |
| **Lymph Node Surgery** |  |  |  | 0.921 |  |  |  |  |  |  |
| None/Unknown | 80 | 864 | 944 |  | 27 | 189 | 216 | 3 | 40 | 43 |
| Lymph nodes removed | 19 | 221 | 240 |  | 4 | 73 | 77 | 0 | 10 | 10 |
| Biopsy of lymph node | 1 | 8 | 9 |  | 0 | 3 | 3 | 0 | 0 | 0 |
| **Radiation Therapy** |  |  |  | 0.734 |  |  |  |  |  |  |
| None/Unknown | 51 | 538 | 589 |  | 18 | 132 | 150 | 2 | 40 | 42 |
| Yes | 49 | 555 | 604 |  | 13 | 133 | 146 | 1 | 10 | 11 |
| **Chemotherapy** |  |  |  | 0.474 |  |  |  |  |  |  |
| No/Unknown | 48 | 484 | 532 |  | 16 | 118 | 134 | 0 | 26 | 26 |
| Yes | 52 | 609 | 661 |  | 15 | 147 | 162 | 3 | 24 | 27 |
| **Brain Metastasis** |  |  |  | 0.037* |  |  |  |  |  |  |
| No/Unknown | 96 | 996 | 1092 |  | 30 | 246 | 276 | 2 | 46 | 48 |
| Yes | 4 | 117 | 121 |  | 1 | 19 | 20 | 1 | 4 | 5 |
| **Liver Metastasis** |  |  |  | 0.001*** |  |  |  |  |  |  |
| No/Unknown | 95 | 888 | 983 |  | 31 | 208 | 239 | 3 | 41 | 44 |
| Yes | 5 | 205 | 210 |  | 0 | 57 | 57 | 0 | 9 | 9 |
| **Lung Metastasis** |  |  |  | <0.001*** |  |  |  |  |  |  |
| No/Unknown | 83 | 569 | 652 |  | 25 | 131 | 156 | 2 | 31 | 33 |
| Yes | 17 | 524 | 541 |  | 6 | 134 | 140 | 1 | 19 | 20 |
| **Laterality** |  |  |  | 0.274 |  |  |  |  |  |  |
| Left | 59 | 573 | 632 |  | 14 | 128 | 142 | 1 | 31 | 32 |
| Right | 41 | 506 | 547 |  | 17 | 131 | 148 | 2 | 17 | 19 |
| Other | 0 | 14 | 14 |  | 0 | 6 | 6 | 0 | 2 | 2 |

Table S5. Distribution of variables according to survival status

| **Characteristic** | **χ2** | **P** |
| --- | --- | --- |
| Age | 52.755 | <0.001*** |
| Race | 0.558 | 0.470 |
| Sex | 4.372 | 0.037* |
| Primary Site | 56.056 | <0.001*** |
| Grade | 50.871 | <0.001*** |
| Histology | 151.190 | <0.001*** |
| Marital Status | 38.181 | <0.001*** |
| Insurance | 0.047 | 0.980 |
| T | 168.947 | <0.001*** |
| N | 152.602 | <0.001*** |
| Surgery | 22.818 | <0.001*** |
| Lymph Node Surgery | 1.631 | 0.44 |
| Radiation Therapy | 10.716 | 0.001*** |
| Chemotherapy | 9.677 | 0.002*** |
| Brain Metastasis | 22.818 | <0.001*** |
| Liver Metastasis | 117.031 | <0.001*** |
| Lung Metastasis | 111.190 | <0.001*** |
| Laterality | 12.104 | 0.002*** |
|  | **Hazard ratio (95%)** |  |
| Tumor Size | 1.001 (1.000,1.001) | <0.001*** |

Table S6. Log-rank tests and Cox analyses of diagnosis of KCBM patients.
